# Pendler Mobil: Die Verwendung von Mobilfunkdaten zur Unterstützung der amtlichen Pendlerstatistik: Können Mobilfunkdaten die amtliche Pendlerrechnung unterstützen?

**DOI:** 10.1007/s11943-021-00294-z

**Published:** 2021-11-12

**Authors:** Sandra Hadam

**Affiliations:** Statistisches Bundesamt, Wiesbaden, Deutschland

**Keywords:** Mobilfunkdaten, Pendlerrechnung, Quelle-Ziel-Matrix, Bewegung, Mobilität, Berufspendler, Mobile network data, Commuter statistics, Origin-destination matrices, Movement, Mobility, Commuter, C81, J60, Y10, Y91

## Abstract

Die Verfügbarkeit von kleinräumigen und aktuellen Pendlerverflechtungen sind für politische wie auch kommunale Entscheidungsfindungen von hoher Bedeutung. Aus dem Pendlerverhalten lassen sich Rückschlüsse auf Arbeitsmarktregionen und die Verteilung der Wohnbevölkerung ziehen, was unter anderem zu einer laufenden Verbesserung der Verkehrsinfrastruktur beiträgt. Die dafür notwendigen Daten veröffentlicht die amtliche Pendlerrechnung. Jedoch weist sie Verbesserungspotenzial im Hinblick auf die zeitliche und räumliche Darstellung der Pendlerverflechtungen von Erwerbstätigen sowie eine fachliche Erweiterung hinsichtlich der Bildungspendler auf.

Dieser Artikel beschreibt die mit dem Projekt *Pendler Mobil *geprüften Erweiterungsmöglichkeiten der amtlichen Pendlerrechnung auf Basis von Quelle-Ziel-Matrizen aus Mobilfunkdaten. Mobilfunkdaten stellen aufgrund ihrer zeitlichen Aktualität und räumlich feinen Auflösung eine robuste Datengrundlage zur flexiblen Abbildung von potenziellen und regelmäßigen Pendlerbewegungen dar. Die potenzielle Leistungsfähigkeit der Mobilfunkdaten ermöglicht damit eine externe Validierung bestehender Pendlerrechnungen oder Pendlerstatistiken sowie eine beiderseitige Ergänzung zur Ermittlung und Darstellungen weiterer Formen des Pendelns der Erwerbsbevölkerung.

Am Fallbeispiel des Bundeslandes Nordrhein-Westfalen werden im Folgenden Gemeinsamkeiten und Unterschiede der übereinstimmenden Pendlerverflechtungen auf Basis von Mobilfunkdaten und der amtlichen Pendlerrechnung erörtert. Dabei gehen wir auf die Herausforderungen der Aufbereitung und Definition geeigneter Mobilfunkdaten durch den Datenanbieter sowie weitere Einflüsse auf die Mobilfunkdaten, wie bspw. durch die zurückgelegte Distanz oder die Verweilzeiten mobiler Aktivitäten, ein. Besonders die Unterschätzung der mobilen Pendlerströme im Vergleich zur amtlichen Pendlerrechnung legt nahe, Modifizierungsansätze der Mobilfunkdaten zu diskutieren. Im Ergebnis können die vorliegenden Mobilfunkdaten potenziell die amtliche Pendlerrechnung durch kleinräumige Pendlerbewegungen in Städten in Form einer erweiterten Zielorts-Bestimmung unterstützen und die Identifizierung von stark frequentierten Arbeitsorten in Städten ermöglichen.

**Zusatzmaterial online:**

Zusätzliche Informationen sind in der Online-Version dieses Artikels (10.1007/s11943-021-00294-z) enthalten.

## Abstract (en)

The availability of small-scale and real-time commuting patterns is of great importance for political as well as communal decision-marking processes. From commuting behaviour, conclusions can be drawn about labour market regions and the distribution of the resident population, which contributes, among other things, to the ongoing improvement of the public transport infrastructure. The data required for this purpose is published by the official commuter statistics. However, it shows potential for improvement with respect to the temporal and spatial representation of commuting patterns of employed persons as well as a technical extension with respect to educational commuters.

This article describes the possibilities for extending the official commuter statistics based on origin-destination matrices from mobile network data, which were investigated by the project *Pendler Mobil*. Due to their temporal timeliness and spatially fine resolution, mobile network data provide a robust data basis for the flexible mapping of potential and regular commuter movements. The potential performance of mobile network data thus enables an external validation of existing commuter calculations or commuter statistics, as well as a two-way complement to identify and represent other forms of commuting by the employed population.

Using the state of North Rhine-Westphalia as a case study, we discuss similarities and differences between corresponding commuting patterns based on mobile network data and the official commuter statistics. In this context, we address the challenges of processing and defining suitable mobile network data by the data provider as well as other influences, such as the distance covered or dwell times of mobile activities on them. Especially the underestimation of mobile commuter flows compared to the official commuter statistics suggests to discuss modification approaches of the mobile network data. As a result, the available mobile network data can potentially support the official commuter statistics by providing small-scale commuter flows in cities as an extended destination determination and enables the identification of highly frequented potential work locations in cities.

## Einleitung

Für diverse politische Entscheidungsfindungen ist es von hoher Bedeutung, Informationen über die Aufenthaltsorte der Bevölkerung zu haben. Durch die regionale Verteilung von Arbeitsplätzen und Wohnorten der Arbeitsbevölkerung entwickeln sich regionale Strukturen, die wiederum durch räumliche Pendlerverflechtungen gekennzeichnet sind (Pütz 2015). Unter Pendlerverflechtungen werden durch die Gesamtheit der zielgerichteten Pendlerbewegungen entstandene Bewegungsmuster verstanden. Das aus Standortentscheidungen resultierende Pendlerverhalten wird weiter durch die Verfügbarkeit und Qualität der vorhandenen Verkehrsinfrastruktur mitbestimmt, wobei die Erreichbarkeit der Standorte ein wichtiges Merkmal der Qualität der Infrastruktur darstellt. Faktoren wie der Zielort, die benötigte Fahrzeit und -entfernung sowie die Erreichbarkeit sind nach Pütz (2015) besonders für Verkehrsplaner von großer Bedeutung, um adäquat auf signifikante Veränderungen in der Verkehrsnutzung und -auslastung reagieren zu können. Eine laufende Verbesserung der Verkehrsinfrastruktur trägt dazu bei, die Erreichbarkeit der Zielorte zu verbessern und den Zeitaufwand des Pendelns zu reduzieren. Weiterhin liefern die Pendlerverflechtungen wichtige Informationen zur Bestimmung von funktionalen Räumen wie Arbeitsmarktregionen oder Stadt-Land-Regionen.

Der Berufsverkehr ist nach Pütz (2015) eine bestimmende Größe in der Inanspruchnahme der Verkehrsinfrastruktur neben anderen Verwendungszwecken wie für Freizeit‑, Erholungs- oder Einkaufsaktivitäten. Da das Pendeln zwischen Wohn- und Arbeitsort nach Haas und Hamann (2008) als „flexible Form der Arbeitskräftemobilität“ an Bedeutung zunimmt, werden die räumlichen Pendlerverflechtungen besonders durch Arbeitsplatzkonzentrationen geprägt (Pütz 2015). Eine grundsätzlich verbesserte räumliche Erreichbarkeit führt folglich zur Bildung oder Verlagerung von Arbeitsplätzen in Regionen, die bis dato an verkehrsungünstig gelegenen Standorten lagen, woraus sich wiederum neue Pendlerverflechtungen ergeben. Um diese Entwicklungen aufzeigen und nachvollziehen zu können, bedarf es zeitlich hochaktueller und räumlich genauer Informationen zu den aktuellen Pendlerverflechtungen.

Sowohl die amtliche Statistik wie auch andere Institutionen decken diesen Bedarf anhand der sogenannten Pendlerstatistik oder auch Pendlerrechnung ab. Die Pendlerrechnung ist eine Sekundärstatistik, die die benötigten Angaben zum Arbeits- und Wohnort der Erwerbsbevölkerung sowie die Merkmale der Pendler aus unterschiedlichen Statistiken bzw. Datenquellen heranzieht. Beispielsweise veröffentlichen der Landesbetrieb Information und Technik Nordrhein-Westfalen (IT.NRW) oder auch die Statistik der Bundesagentur für Arbeit (BA) jährlich die aktuellen Pendlerverflechtungen in Form eines interaktiven Pendleratlanten. Durch die unterschiedliche Datenlage in beiden Angeboten besteht jedoch eine zeitliche Verzögerung bis zur Veröffentlichung der Ergebnisse. Die BA verwendet beispielsweise ihre eigene Beschäftigungsstatistik mit mehr als 30 Mio. Datenpunkten basierend auf aktuellen Daten aus dem Meldeverfahren zur Sozialversicherung und ermöglicht dadurch eine Veröffentlichung der Ergebnisse mit einem ca. einjährigen Zeitverzug. Allerdings beinhaltet die Pendlerstatistik der BA nur die sozialversicherungspflichtig Beschäftigten (Bundesagentur für Arbeit 2020). Darauf aufbauend enthält auch die Pendlerrechnung von IT.NRW diese Daten, welche jedoch mit weiteren Informationen aus dem Mikrozensus sowie der Personalstandstatistik zu weiteren Erwerbstätigen unterfüttert werden, wodurch der Informationsgehalt gesteigert wird. Durch die Kombination mehrerer Datenquellen resultiert in den Ergebnissen bei der Veröffentlichung jedoch ein zweijähriger Zeitverzug. Weiterhin findet die räumliche Auflösung nur bis auf die Kreis- oder Gemeindeebene statt. Insgesamt offenbaren diese beiden Beispiele zeitliche und räumliche Verbesserungsmöglichkeiten in der Wiedergabe und Darstellung der aktuellen Pendlerverflechtungen. Besonders eine Verbesserung bzw. Weiterentwicklung der Pendlerrechnung der amtlichen Statistik – hierbei ist exemplarisch die Pendlerrechnung von IT.NRW zu nennen – ist für die Statistischen Ämter des Bundes und der Länder von Bedeutung.

Diverse Studien und Forschungsarbeiten haben sich daher mit der Frage beschäftigt, inwieweit alternative Datenquellen zeitlich hochaktuelle und räumlich genaue Mobilitätsanalysen der Bevölkerung zulassen. Mobilfunkdaten, sogenannte Signaldaten, bieten eine vielversprechende Datenquelle. Hierbei werden alle Signale im entsprechenden Mobilfunknetz vom Netzbetreiber erfasst. Sie werden automatisch erzeugt, sofern das mobile Endgerät nicht ausgeschaltet ist oder sich im Flugmodus befindet und registrieren lediglich die Ortsangabe des Funkmastes, mit dem das mobile Endgerät zu einem bestimmten Zeitpunkt verbunden ist. Aufgrund der hohen Penetrationsrate von Nutzerinnen und Nutzern mobiler Endgeräte in der Bevölkerung (Statistisches Bundesamt 2020) und der zeitlich und räumlich genauen Verortung dieser Geräte lassen sich vielversprechende Fragestellungen zur Mobilität der Bevölkerung betrachten.

Im Zuge erster Machbarkeitsstudien hat das Statistische Bundesamt bereits anhand von Korrelationsmodellen den Zusammenhang zwischen statischen Mobilfunkdaten und amtlichen Bevölkerungszahlen für das Bundesland Nordrhein-Westfalen (NRW) auf kleinräumiger Ebene der Gitterzellen herstellen können (Hadam et al. 2020a). Die zeitliche Aktualität und Kleinräumigkeit spiegelt sich dabei auch in der Dynamik der Mobilfunkdaten wider und in der Fragestellung, wohin sich die Mobilfunkaktivitäten im Zeitverlauf bewegen. Wird diese Fragestellung auf die amtliche Statistik projiziert, so kann anhand dieser Daten erforscht werden, ob das Pendlerverhalten und die Pendlerverflechtungen der erwerbstätigen Bevölkerung anhand von Mobilfunkdaten abgebildet werden kann. Deville et al. (2014) zeigen bereits anhand von Mobiltelefondaten, sogenannten Call Detail Records (CDRs), auf, wie dynamisch die Verteilung einer Bevölkerung im Zeitverlauf ist. CDRs liefern individuelle Informationen zu Mobiltelefonnutzenden auf einer hohen räumlichen Auflösung, welche im Gegensatz zu den Signaldaten jedoch ereignisbasiert sind. Die CDRs sind daher nur verfügbar, wenn der Telefonnutzende bspw. aktiv einen Anruf tätigt oder eine SMS bzw. mobile Daten sendet (Jacques 2018). Am Beispiel Frankreichs und Portugals stellen Deville et al. (2014) mit diesen Daten die Verlagerung der jeweiligen Bevölkerung an die Küstenregionen in den Sommermonaten präzise dar. Diese Informationen sind aktuell auch im Fall von Katastrophen oder Epidemien entscheidend. Ein Beispiel stellen die Auswirkungen der Covid-19 Pandemie auf die Bewegungsfreiheit und damit die Mobilität der Bevölkerung dar. Anhand von Mobilfunkdaten kann das Mobilitätsverhalten der Bevölkerung tagesaktuell ausgewertet und Beschränkungsmaßnahmen können auf ihre Wirkung geprüft werden (Statistisches Bundesamt 2021a). Unabhängig davon betrachten De Jonge et al. (2012), inwieweit sich Bewegungsmuster in den Niederlanden aus CDRs für die amtliche Statistik ableiten lassen und vergleichen diese mit der niederländischen Mobilitätserhebung, genauer dem Verkehrsaufwand nach Verkehrsmittel in den Niederlanden.^1^ Sie berechnen dabei die Reisedistanzen der Mobiltelefonnutzenden und verwenden diesen als Näherungswert für die Mobilitätsstatistik. Aufgrund der Limitation der enthaltenen Informationen, die mit den CDR Daten einhergehen, werden die Anzahl der Bewegungen sowie die gesamten Mobilitätsverflechtungen aus den CDRs mitunter unterschätzt oder nicht flächendeckend abgebildet. Im Zusammenhang mit den CDR Daten wird oftmals auch verstärkt die wirtschaftliche Aktivität für räumliche Regionen – wie bspw. Arbeitsmarktregionen – abgeleitet, indem die Intensität und damit das Volumen von aktiven Mobiltelefonen als Indikator für wirtschaftliche Aktivität interpretiert wird (Arhipova et al. 2020). Novak et al. (2013) wiederum betrachten Pendlerströme, worunter aggregierte zielgerichtete Pendlerbewegungen zu verstehen sind, anhand von Standortdaten der Mobiltelefone in Estland. Die Standortdaten bilden in ihrer Studie die wichtigsten Pendlerströme ab und identifizieren dabei wichtige regionale Zentren. Inwieweit kleinräumige und zeitlich aktuelle Pendlerdaten in Verknüpfung mit anderen Datenquellen weiterführend genutzt werden können, zeigen Hadam et al. (2020b). Sie nutzen die Intensitäten und die Verteilung der Mobilfunkdaten, um eine alternative Erwerbslosenquote für funktionale städtische Gebiete anhand des Arbeitsortes und des Pendlerverhaltens der Erwerbspersonen zu berechnen.

Das hier beschriebene Projekt *Pendler Mobil* wird in Kooperation mit IT.NRW durchgeführt und sieht Analysen zur Bevölkerungsmobilität in NRW auf Basis von Mobilfunkdaten aus dem Netz der Deutschen Telekom vor. Fortführend wird das Ziel verfolgt, Bereiche anhand des Beispiels von NRW zu identifizieren, in denen Mobilfunkdaten zu einer bundesweiten Ergänzung der bisherigen Pendlerstatistik bzw. -rechnung beitragen können. Anhand von Quelle-Ziel-Matrizen wird untersucht, ob Daten aus dem Mobilfunknetz genutzt werden können, um Pendlerströme im Tagesverlauf abzubilden und inwieweit die aktuelle Pendlerrechnung für NRW mit diesen Daten ergänzt werden kann. Ein Vorteil der Nutzung von Mobilfunkdaten zur Unterstützung der Pendlerrechnung liegt mitunter darin, dass Mobilfunkdaten robust gegenüber Unschärfen bei der Betriebsstättenzuordnung sind. Der Zielort in den Mobilfunkdaten entspricht dem realen potenziellen Arbeitsplatz und damit einhergehenden existenten Pendlerverflechtungen. Letztere sind dadurch nicht abhängig vom eingetragenen Hauptsitz des Unternehmens, welcher bei der amtlichen Pendlerrechnung durch die entsprechenden Datenquellen zwar hinterlegt ist, aber zu dem de facto nicht gependelt wird. Eines der wesentlichsten Vorhaben besteht folglich darin, die arbeitende Bevölkerung und daraus die Pendler aus den Mobilfunkdaten abzuleiten, um diese unter anderem mit der amtlichen Pendlerrechnung von IT.NRW vergleichen zu können. Da die Pendlerrechnung von IT.NRW, wie auch generell, nur auf Gemeindeebene zur Verfügung gestellt wird und aufgrund der dafür notwendigen Daten jährlich mit einem Zeitverzug der Ergebnisse von zwei Jahren aktualisiert werden kann, wird ergänzend geprüft, ob Mobilfunkdaten zu einer kleinräumigeren und schnelleren Abbildung von Pendlerströmen in Form einer *experimentellen Pendlerrechnung* beitragen können. Die Pendlerrechnung dient hierbei als Vergleichsmaßstab, um die Plausibilität der aufbereiteten Mobilfunkdaten anhand dieser zu überprüfen.

Der Artikel ist wie folgt gegliedert: Im nachfolgenden Abschnitt wird die Datengrundlage beschrieben und auf die amtliche Pendlerrechnung sowie mögliche Ergänzungen bzw. Erweiterungsmöglichkeiten dieser eingegangen. Zudem werden die hierfür benötigten Mobilfunkdaten sowie ihre Aufbereitung anhand der Pendlerrechnung vorgestellt, um die Daten anschließend in Abschn. 3 mit den amtlich ermittelten Pendlerströmen zu validieren. Weiterführend werden kleinräumige Pendlerbewegungen für eine erweiterte Zielorts-Bestimmung in Städten hergeleitet und Zusammenhänge zwischen Berufspendlern nach Beschäftigungsumfang und den Mobilfunkdaten geknüpft. In Abschn. 4 diskutieren wir die zuvor ermittelten Ergebnisse und gehen auf mögliche Einflüsse auf die Pendlerbewegungen in den Mobilfunkdaten ein. Weiterhin werden Modifizierungsansätze in der Definition und Erstellung der Mobilfunkdaten diskutiert. Im letzten Abschnitt wird ein Fazit des Erweiterungspotenzials der amtlichen Pendlerrechnung durch Mobilfunkdaten gezogen und weitere Schlussfolgerungen zur Diskussion gestellt.

## Datengrundlage

### Die amtliche Pendlerrechnung und ihre Erweiterungsmöglichkeiten

Die amtliche Pendlerrechnung dient zur Ermittlung der täglich zur Arbeit pendelnden Personen, um damit flächendeckende Angaben zur pendelnden Bevölkerung zu ermöglichen. Als Pendler^2^ werden erwerbstätige Personen verstanden, die einen Arbeitsweg vom Wohn- zum Arbeitsort zurücklegen. Die Bedingung hierbei ist, dass sich der Arbeitsort vom Wohnort unterscheiden muss, wobei sich der Arbeits- und Wohnort für gewöhnlich auf die räumliche Gebietsstruktur der Gemeinden bezieht (IT.NRW 2020; Bundesagentur für Arbeit 2021). Als erwerbstätig werden im Folgenden, entsprechend der Definition der internationalen Arbeitsorganisation (ILO), alle Personen im arbeitsfähigen Alter verstanden, die eine oder mehrere gegen Entgelt ausgerichtete Tätigkeiten ausüben, unabhängig von der Dauer oder dem Umfang der tatsächlich geleisteten Tätigkeit (International Labour Organization 2013).^3^

In der Pendlerrechnung der amtlichen Statistik werden zu den Erwerbstätigen alle sozialversicherungspflichtig Beschäftigten gezählt, deren Grundlage die Beschäftigungsstatistik der BA bildet (IT.NRW 2020; Bundesagentur für Arbeit 2021).^4^ Die Pendlerstatistik der BA verwendet bspw. nur ihre eigene Beschäftigungsstatistik als Datengrundlage. Der daraus resultierende interaktive Pendleratlas gibt somit Informationen aller sozialversicherungspflichtig pendelnden Beschäftigten für jeden Kreis in Deutschland des Vorjahres zum Stichtag 30. Juni wieder, wobei die zehn größten Pendlerströme aus den nahegelegensten Kreisen mit einer Entfernung von ca. 150 km ausgewiesen werden.^5^

Aufgrund einer speziellen Landesstatistik in den Bundesländern Baden-Württemberg, Hessen und NRW wird eine Pendlerrechnung entsprechend nur durch diese drei Statistischen Landesämter anhand einer abgestimmten Methodik der Statistischen Ämter der Länder erstellt und veröffentlicht. Daher liegt bislang keine bundesweite Pendlerrechnung der Statistischen Ämter des Bundes und der Länder vor. In den Pendlerrechnungen der genannten Statistischen Landesämter werden neben den sozialversicherungspflichtig Beschäftigten und geringfügig Beschäftigten weiterhin Beamtinnen und Beamte, Richterinnen und Richter aus der Personalstandstatistik sowie die Selbstständigen und mithelfenden Familienangehörigen aus dem Mikrozensus hinzugezählt (IT.NRW 2020; Dettmer und Emmel 2018; Statistisches Landesamt Baden-Württemberg 2019b). Damit bauen die Pendlerrechnungen der Statistischen Landesämter auf identischen Datengrundlagen auf. IT.NRW veröffentlicht bspw. jährlich die Pendlerbewegungen der Erwerbstätigen auf Gemeinde- und Kreisebene mit einem zweijährigen Zeitverzug und stellt diese für alle Erwerbstätigen in NRW in einem interaktiven Pendleratlas dar (IT.NRW 2020).^6^ Neben den interaktiven Pendleratlanten von IT.NRW und der Statistik der BA wurden die Pendlerrechnungen – jedoch nicht in Form einer interaktiven, d. h. jährlich aktualisierten Karte – vom Statistischen Landesamt Baden-Württemberg und vom Hessischen Statistischen Landesamt in entsprechenden Veröffentlichungen für die jeweiligen Bundesländer publiziert (siehe hierzu Statistisches Landesamt Baden-Württemberg 2019a; Dettmer und Emmel 2018).

Deutschlandweit wird die Pendlerstatistik nur auf Basis des Mikrozensus im Rahmen eines vierjährigen Zusatzprogramms ermittelt und vom Statistischen Bundesamt veröffentlicht, wobei die Beantwortung der Fragen zum Pendlerverhalten freiwillig ist. Die Auswertungen geben den Bundesdurchschnitt der pendelnden Erwerbstätigen, nachfolgend Berufspendler genannt, nach der Stellung im Beruf, der Entfernung, dem Zeitaufwand und dem benutzten Verkehrsmittel für den Hinweg zur Arbeitsstätte wieder (Statistisches Bundesamt 2017a, b). Aktuell liegen die damit ermittelten Angaben der Berufspendler aus dem Mikrozensus für das Jahr 2016 vor.^7^

Berufspendler werden als übergemeindliche Pendler definiert, sofern ihr Arbeitsort nicht in derselben Gemeinde wie ihr Wohnort liegt. Andernfalls spricht man von innergemeindlichen Pendlern. Übergemeindliche Pendler kategorisiert man weiterhin nach Ein- und Auspendlern. Einpendler sind erwerbstätige Personen, die nicht in ihrer Arbeitsgemeinde wohnen, wohingegen Auspendler nicht in der Gemeinde arbeiten, in der sie wohnen (IT.NRW 2020; Bundesagentur für Arbeit 2021). Zum besseren Verständnis sei als Beispiel Person *i* wohnhaft in Gemeinde A und berufstätig in Gemeinde B angeführt. D. h., Person *i* pendelt von Gemeinde A nach Gemeinde B und umgekehrt. Aus Sicht von Gemeinde A ist Person *i *demnach ein Auspendler, da Person *i *außerhalb ihres Wohnortes arbeitet und sich damit aus ihrem Wohnort rausbewegt. Aus Sicht von Gemeinde B ist Person *i* hingegen ein Einpendler, da diese in einer Gemeinde arbeitet, in der sie nicht wohnt und damit in Gemeinde B einpendelt bzw. sich reinbewegt.

Zusätzliche Differenzierungen der Berufspendler werden unter anderem nach den Merkmalen Alter, Geschlecht, Beschäftigungsumfang oder dem Wirtschaftsbereich vorgenommen. Dabei wird die Strecke des Pendelweges bzw. die Distanz zwischen dem Wohn- und Arbeitsort für die Plausibilisierung der Pendlerströme verwendet. Die Distanz wird durch die Luftlinienentfernung zwischen den geografischen Mittelpunkten des Arbeits- und Wohnortes berechnet. Ein Pendelweg wird hierbei als plausibel bewertet, sofern die berechnete Distanz zwischen Wohn- und Arbeitsort die Grenze von 80 km nicht überschreitet und diese damit noch als täglich zu bewerkstelligen eingestuft wird (IT.NRW 2020).

Insgesamt bildet die Pendlerrechnung von IT.NRW das Pendlerverhalten von ungefähr 91 % der Erwerbstätigen in NRW ab.^8^ Da die Qualität der Pendlerrechnung von IT.NRW insgesamt als sehr gut zu bewerten ist (IT.NRW 2020), wird im Folgenden nicht die Qualität der veröffentlichten Pendlerströme, sondern potenzielle Erweiterungsmöglichkeiten der amtlichen Pendlerrechnung in NRW für das aktuell veröffentlichte Jahr 2019 an diesem Fallbeispiel betrachtet. Bundesweite Ergänzungen werden zum einen in der Darstellung der Pendlerverflechtungen in räumlichen Gebieten unterhalb der Gemeindeebene gesehen. Besonders kleinräumige Pendlerverflechtungen in großen Städten liefern wertvolle Informationen zu stark frequentierten städtischen Bereichen oder bis dahin nicht aufgezeigten Arbeitsplatzkonzentrationen. Auch im Hinblick auf die Infrastruktur des öffentlichen Nahverkehrs bieten kleinräumige Informationen eine größere und vor allem genauere Planungsmöglichkeit bei der effizienten Gestaltung. Zudem lassen sich ausschließlich anhand kleinräumiger Angaben zum Wohn- und Arbeitsort innergemeindliche Verflechtungen sichtbar machen.

Da auch die Aktualität der von IT.NRW veröffentlichten Pendlerrechnung des Jahres 2019 aufgrund der verschiedenen Datenquellen Verbesserungspotenzial aufzeigt, wird auch eine mögliche Unterstützung in der zeitlichen Komponente durch die nachfolgend beschriebenen Mobilfunkdaten diskutiert. Zum anderen besteht eine weitere Ergänzungsmöglichkeit in der Abbildung von Bildungspendlern, worunter Schülerinnen und Schüler sowie Studierende zu verstehen sind,^9^ da diese aufgrund von Dateninkonsistenzen bei den Wohnortangaben in der Pendlerrechnung nicht berücksichtigt werden (IT.NRW 2020).^10^ Wegen der aktuell begrenzten Möglichkeiten ausschließlich Bildungspendler aus den Mobilfunkdaten abzuleiten, fokussieren wir uns in diesem Artikel primär auf die Darstellung der Berufspendler.^11^ Dennoch besteht der Bedarf, regional tiefere Verflechtungen der Bildungspendler zu ermitteln, wie die Zahlen des Statistischen Bundesamtes (2017c) zu den Bildungspendlern nach Entfernung, Zeitaufwand und benutztem Verkehrsmittel für den Hinweg zur Schule oder Hochschule für das Jahr 2016 andeuten. Insbesondere zeigt der hohe Anteil von ca. 47 % der Bildungspendler, die regelmäßig den öffentlichen Personenverkehr nutzen und auch beanspruchen, wie relevant kleinräumige und aktuelle Angaben zum Pendlerverhalten dieser Personengruppe für eine Optimierung sowie Quantifizierung der Auslastung der Verkehrsinfrastruktur ist.

### Mobilfunkdaten: Datendefinition und -aufbereitung

Mobilfunkdaten können je nach Spezifikation zahlreiche Strukturen annehmen und damit diverse Fragestellungen beantworten. Daher ist es essentiell die Daten entsprechend dem Forschungsziel aufzubereiten. Ziel dieser Arbeit muss es sein, die Berufspendler bestmöglich aus den Mobilfunkdaten abzuleiten, um diese mit der amtlichen Pendlerrechnung 2019 vergleichen zu können.

Das Statistische Bundesamt und IT.NRW nutzen Quelle-Ziel-Matrizen aus dem Netz der Deutschen Telekom, um Mobilitätsanalysen der Bevölkerung mit besonderem Fokus auf die Pendlerströme in NRW durchzuführen. Quelle-Ziel-Matrizen stellen dabei anonymisierte und aggregierte Bewegungen von Signalen bzw. mobilen Aktivitäten im Mobilfunknetz vom Start- zum Zielort (daher Quelle-Ziel-Matrix), im Folgenden auch als Bewegungsverflechtungen bezeichnet, dar. Unter einer mobilen Aktivität wird ein Signal an einem Funkmast verstanden, welches durch eine Mindestverweildauer des mobilen Endgerätes in einem Untersuchungsgebiet bedingt wird. Das mobile Endgerät darf dabei nicht ausgeschaltet oder im Flugmodus sein. Im Gegensatz zu CDR Daten sind bei den hier vorliegenden Signaldaten keine Informationen zur durchgeführten Aktivität, wie getätigte Anrufe, SMS, mobile Datennutzung o. ä., enthalten.

**Figure 1:**
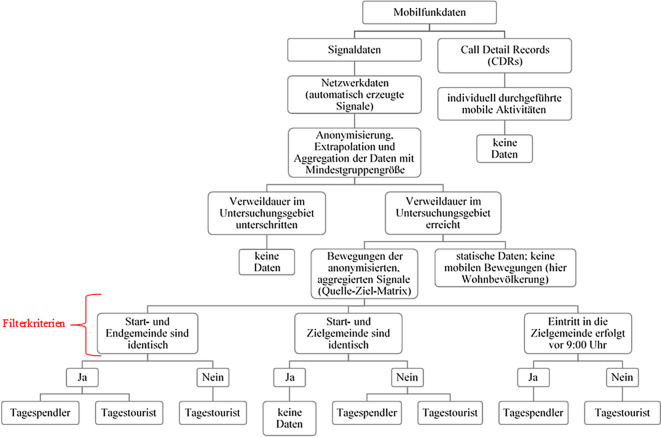

Die Daten enthalten weiterhin tägliche und extrapolierte Bewegungsverflechtungen für die Monate August, September und Oktober aus dem Jahr 2019 mit einer Verweildauer von zwei Stunden am Zielort, im Folgenden auch Untersuchungsgebiet genannt. Die Verweildauer gibt an, wie lange sich das mobile Endgerät durchschnittlich in einem Untersuchungsgebiet aufhalten muss, um als Aktivität gezählt zu werden. D. h., alle mobilen Aktivitäten, die weniger als zwei Stunden im Untersuchungsgebiet aktiv waren, sind in den vorliegenden Mobilfunkdaten nicht enthalten. Alle Aktivitäten, die länger als zwei Stunden im Untersuchungsgebiet aktiv waren, sind auch in den Daten erhalten. Abb. 1 stellt die beschriebene Datenlage schematisch dar.

Das Untersuchungsgebiet stellt in diesem Artikel generell eine Gemeinde^12^ dar. Hierbei werden Städte mit mehr als 100.000 Einwohnern^13^ zusätzlich in Gitterzellen mit Gitterweiten von 250 × 250 m bis zu 4 × 4 km, abhängig vom Mobilfunknetz des Anbieters, unterteilt. Die aggregierten Mobilfunkdaten wurden weiterhin – aufgrund datenschutzrechtlicher Regelungen – erst ab einer Mindestgruppengröße von fünf mobilen Aktivitäten an das Statistische Bundesamt und IT.NRW übermittelt. Durch das Anonymisierungsverfahren des Datenanbieters können die Bewegungsverflechtungen darüber hinaus lediglich tageweise (innerhalb von 24 h) nachvollzogen werden (Bundesbeauftragte für den Datenschutz und die Informationsfreiheit 2017), weshalb auch nachfolgend der Begriff „Tagespendler“ in den Mobilfunkdaten Verwendung findet. Eine Validierung der Bewegungsverflechtungen durch die Betrachtung längerer, ununterbrochener Zeitperioden ist daher nicht möglich. D. h., es kann nicht nachvollzogen werden, ob die identifizierten Bewegungen täglich bzw. regelmäßig stattfinden oder diese ggf. nur Ausnahmeerscheinungen sind. Auch erlaubt die Auswertung der anonymisierten mobilen Aktivitäten keinen direkten Rückschluss auf die Ursache der ermittelten Bewegung.

Um sicherzustellen, dass vor allem die Berufspendler in den Mobilfunkdaten enthalten sind, wurden weiterhin Bedingungen bzw. Filterkriterien formuliert, die sich an der zuvor erläuterten Definition eines Berufspendlers orientieren. Die mobilen Aktivitäten in den Mobilfunkdaten unterliegen somit folgenden Bedingungen (vgl. Abb. 1):

- Die Start- und Endgemeinde sind identisch. Berufspendler starten an ihrem Wohnort und kehren üblicherweise an denselben zurück, weshalb das erste und das letzte Signal des Tages eines mobilen Endgerätes in der gleichen Gemeinde und damit dem potenziellen Wohnort verzeichnet werden müssen.
- Die Start- und Zielgemeinde sind nicht identisch. Damit wird die Bedingung gestellt, dass die mobilen Aktivitäten in der Zielgemeinde bzw. dem Untersuchungsgebiet – demnach am potenziellen Arbeitsort – außerhalb der potenziellen Wohnsitzgemeinde stattfinden und sich folglich aus der Wohnsitzgemeinde entfernen müssen. Dadurch kann gewährleistet werden, dass nur übergemeindliche Bewegungen in den Mobilfunkdaten enthalten sind. Weiterhin lässt sich daraus interpretieren, dass eine Person mit einem mobilen Endgerät, die ihr erstes und letztes Mobilfunksignal des Tages außerhalb des Untersuchungsgebietes tätigt, im Untersuchungsgebiet arbeitet und nach der Pendlerdefinition im Untersuchungsgebiet als Einpendler angesehen wird.
- Eintritt in die Zielgemeinde erfolgt vor 9:00 Uhr. Für eine trennschärfere Unterscheidung zwischen Tagespendlern und Bewegungen zu anderen Zwecken, wie bspw. Freizeit- oder touristische Aktivitäten, wird weiterhin angenommen, dass Berufspendler früher am Tag ihre Wohngemeinde verlassen als Nicht-Berufspendler.

Zusätzlich wird zur Bestimmung des Wohnortes behelfsmäßig die Vertragspostleitzahl neben dem ersten und letzten Signal des Tages durch den Datenanbieter verwendet, sofern diese zur Verfügung steht. Ebenfalls werden nur die nationalen SIM-Karten in den Daten beibehalten, um Verzerrungen durch touristische Aktivitäten von ausländischen Tagestouristen zu minimieren.

Insgesamt stellt die letzte Bedingung (c) der Filterkriterien jedoch eine besondere Schwachstelle in den Daten bzw. der schwierigen Datenspezifikation dar, da dadurch zwangsweise Schichtarbeitende, Teilzeitbeschäftigte oder auch mobile Arbeitskräfte, nicht oder nur marginal einbezogen werden.

Aufgrund der schwierigen Eingrenzung der Pendlerbewegungen in den vorliegenden Mobilfunkdaten finden sich daher neben den Tagespendlern folglich zwei weitere Personenkategorien, die Tagestouristen^14^ und die Wohnbevölkerung, die im Wesentlichen die Bewegungsverflechtungen der Nicht-Berufspendler abdecken. Die sogenannten Tagestouristen weisen hierbei ein ähnliches Verhalten wie die Tagespendler in den Mobilfunkdaten auf und sind daher schwierig voneinander abzugrenzen, wie in Abb. 1 anhand der umgesetzten Filterkriterien offensichtlich wird. Die grundlegende Unterscheidung beider Personenkategorien ist das Ziel bzw. die Motivation der Bewegung, welche beim Tagestouristen auf Freizeit- oder touristische Aktivitäten zurückzuführen ist. Da der Zielort der mobilen Bewegungen allein jedoch nicht zu belastbaren Aussagen führt, werden weitere Unterscheidungsmerkmale der Personenkategorien eingeführt. Anders als der Tagespendler muss nach der Definition des Datenanbieters ein Tagestourist nicht wieder an seinen Wohnort zurückkehren (vgl. Abb. 1). Zudem tritt der Tagestourist Annahme bedingt erst nach 9:00 Uhr Lokalzeit in die Zielgemeinde ein. Dies trifft sicherlich auch auf Berufspendler zu, allerdings stellt diese Bedingung, wie in der schematischen Darstellung in Abb. 1 klargestellt wird, das einzige Filterkriterium dar, das konsequent zwischen Tagespendler und Tagestourist unterscheidet.

Weiterhin wird die Wohnbevölkerung in den Daten ausgewiesen, die jedoch keine Bewegung aufzeigt, da grundsätzlich nur der Wohnort als Untersuchungsgebiet betrachtet wird und folglich kein Wechsel der Gemeinde erfolgen darf. Dementsprechend werden sie in der folgenden Arbeit nicht weiter berücksichtigt.

Um anhand der gefilterten Mobilfunkdaten im nachfolgenden Abschnitt Vergleiche mit der Pendlerrechnung von IT.NRW des Jahres 2019 durchführen zu können, müssen die Daten zeitlich und räumlich weiter aufbereitet werden. Hierzu werden zum einen nur ferienfreie Werktage aus den Monaten August, September und Oktober 2019 aus den Mobilfunkdaten verwendet, da Ferienzeiten und Feiertage die alltäglichen Muster des Pendlerverhaltens im Regelfall nicht adäquat wiedergeben. Zum anderen müssen die regionalen Gebiete in den Mobilfunkdaten denen der Pendlerrechnung angepasst werden, um unter anderem die Distanzen zwischen Start- und Zielgemeinde entsprechend der Pendlerrechnung zu berechnen. Das bedeutet, dass die Startorte der mobilen Bewegungen, welche in den Mobilfunkdaten auf Ebene der 5‑stelligen Postleitzahlen vorliegen, den überdeckenden Gemeinden zugeordnet werden müssen.

Des Weiteren werden die täglichen Bewegungsverflechtungen zu einem Durchschnittswert zusammengefasst. So kann annäherungsweise gewährleistet werden, dass die regulären Pendlerbewegungen in den Mobilfunkdaten stärker einbezogen werden. Dafür werden zunächst alle Pendlerströme mit Zielorten auf Ebene der Gitterzelle – sofern vorhanden – pro Tag auf die zugehörige Gemeinde aufaggregiert und daraus ein Durchschnittswert der Bewegungsverflechtungen aller verbliebenen Auswertungstage für alle enthaltenen Kombinationen von Start- und Zielgemeinde gebildet.^15^ Für die Angabe der Distanzen zwischen diesen Start- und Zielgemeinden in den Mobilfunkdaten wird die Entfernung der geografischen Koordinaten (ETRS89) der Gemeindemittelpunkte unter Verwendung des Satzes des Pythagoras in Kilometern berechnet.^16^

Für die Zusammenführung der aufbereiteten Mobilfunkdaten mit der Pendlerrechnung werden anschließend die einzelnen Start-Ziel-Verbindungen in beiden Datenquellen mit einer eindeutigen selbsterstellten Kennung versehen, um diese im nachfolgenden Abschn. 3.1 miteinander verknüpfen und vergleichen zu können.

## Mobilfunkdaten in der amtlichen Pendlerrechnung

### Vergleich der Pendlerbewegungen auf Basis von Mobilfunkdaten mit der amtlichen Pendlerrechnung

Bevor Möglichkeiten zur Anreicherung der amtlichen Pendlerrechnung mit den vorliegenden Mobilfunkdaten diskutiert werden können, müssen wir zunächst ermitteln, inwieweit die Quelle-Ziel-Matrizen der Mobilfunkdaten 2019 mit denen der Pendlerrechnung 2019 übereinstimmen. Durch eine räumliche Zuordnung aller Pendlerverflechtungen in beiden Datenquellen anhand der eindeutigen Kennung ist es möglich, all jene Pendlerbewegungen aus den Mobilfunkdaten zu extrahieren, die in der Pendlerrechnung von IT.NRW ausgewiesen werden und sie dementsprechend zu validieren.

Hierfür bedarf es einer geeigneten Mobilfunkdatenbasis. Da eine trennscharfe Abgrenzung der einzelnen Personengruppen und damit der Pendlerverflechtungen durch die zuvor in Abschn. 2.2 beschriebenen Filterkriterien jedoch nur approximativ möglich ist, stehen zur Abbildung der Pendlerverflechtungen zwei Optionen zur Verfügung. Entweder es werden nur die Tagespendler einbezogen, womit eine belastbare Datenbasis für die Darstellung der Pendlerverflechtungen der potenziellen Berufspendler aus den Mobilfunkdaten gebildet wird. Dadurch wird allerdings ein Informationsverlust an Bewegungsverflechtungen entstehen, da diese Daten durch die in Abschn. 2.2 diskutierten Filterkriterien sehr stark selektiert sind. Alternativ können alle Personenkategorien verwendet werden, das schließt die Tagespendler, die Tagestouristen sowie die Wohnbevölkerung ein, wodurch deutlich mehr Bewegungsverflechtungen bzw. mobile Aktivitäten in den Daten enthalten sind. In diesem Fall geht hingegen die Möglichkeit verloren, charakteristischere Aussagen zu den Berufspendlern zu treffen. Tab. 1 illustriert diesen Zielkonflikt anhand des Pearson-Korrelationskoeffizienten für die mit der Pendlerrechnung 2019 übereinstimmenden mobilen Pendlerströme auf Basis der Tagespendler sowie aller drei Personenkategorien. Der Pearson-Korrelationskoeffizient ermittelt den linearen Zusammenhang bzw. die Stärke des Zusammenhangs zwischen den gemeinsamen Pendlerströmen in den Mobilfunkdaten und der Pendlerrechnung.

**Table 1:** 

Übereinstimmende Pendlerströme	r	t‑Wert	df	Pr(> |t|)	∑ Mobilfunkbewegungen
Tagespendler	0,846	233,6	21.698	<2.2e-16	28.509
Alle Personenkategorien	0,906	368,73	29.839	<2.2e-16	89.093

Im Ergebnis liegt die Korrelation der übereinstimmenden Pendlerströme beider Datenquellen, unter ausschließlichem Einbezug der Tagespendler, bei 0,85 (vgl. Tab. 1) und deutet damit einen sehr starken positiven linearen Zusammenhang zwischen den Pendlerströmen beider Datenquellen an. Weiterhin wird ersichtlich, dass die Einbeziehung aller Personenkategorien zu einer moderaten Zunahme der Korrelation führt und damit der zuvor diskutierte und erwartete höhere Informationsgehalt zum Tragen kommt. Dies wird neben dem Korrelationskoeffizienten in Höhe von 0,91 auch bei der gestiegenen Anzahl an Freiheitsgraden (df) sichtbar.

Betrachten wir weiterhin die gesamten Mobilfunkbewegungen (∑ Mobilfunkbewegungen) in Tab. 1, ohne Verbindung zur Pendlerrechnung, stehen uns unter Einbezug aller Personenkategorien rund 89.000 Mobilfunkbewegungen zur Verfügung und mit den Tagespendlern nur rund 28.500 Mobilfunkbewegungen. Der Einbezug aller Personenkategorien beinhaltet offensichtlich Mobilitätsverflechtungen, die überwiegend nicht den Pendlerverflechtungen und stattdessen eindeutig touristischen Aktivitäten zuzuordnen sind, wie der Besuch von Naherholungsorten, Einkaufstandorten oder Veranstaltungen. Bei dieser Datenauswahl würde außerdem die Möglichkeit verloren gehen, weitere Berufspendlerströme aus Mobilfunkdaten herzuleiten, welche möglicherweise noch nicht in der Pendlerrechnung erfasst wurden, da Aussagen hierzu aufgrund der Vermischung mit anderweitigen Mobilitätsverflechtungen nicht mehr getroffen werden können. Daher werden beim Einbezug aller Personenkategorien keine gerechtfertigten oder belastbaren Erweiterungsmöglichkeiten der Pendlerrechnung gesehen, wie eine charakteristische kleinräumige Zielorts-Bestimmung der Berufspendler (siehe Abschn. 3.2).

Da das Ziel dieser Arbeit die fachliche Unterstützung der amtlichen Pendlerrechnung bzw. eine Prüfung der Möglichkeiten hierfür ist, sollen möglichst plausible Pendlerwege in den Daten ermittelt werden. Daher werden für die nachfolgenden Analysen ausschließlich die Tagespendler aus den Mobilfunkdaten verwendet.^17^ Insgesamt erhalten wir somit 21.700 übereinstimmende Pendlerströme aus beiden Datenquellen durch die eindeutige Verknüpfung der jeweils gelisteten Start-Ziel-Verbindung. Zur Einordnung der Größenordnung liegen ca. 44.600^18^ amtlich erfasste Pendlerbewegungen und insgesamt 28.509 Mobilfunkbewegungen (siehe ∑ Mobilfunkbewegungen in Tab. 1) in der Analyse vor. Ungefähr 22.900 Pendlerverflechtungen aus der amtlichen Pendlerrechnung stimmen damit nicht mit den Mobilfunkdaten überein oder sind nicht in den Mobilfunkdaten enthalten.^19^ Mit der ausschließlichen Nutzung der Tagespendler liegen in den Mobilfunkdaten andererseits, durch die Differenz aller Mobilfunkbewegungen der Tagespendler und der mit der Pendlerrechnung übereinstimmenden Bewegungen (vgl. Tab. 1), 6809 potenzielle Pendlerverflechtungen vor, die bislang noch nicht durch die amtliche Pendlerrechnung abgedeckt werden.^20^

**Figure 2:**
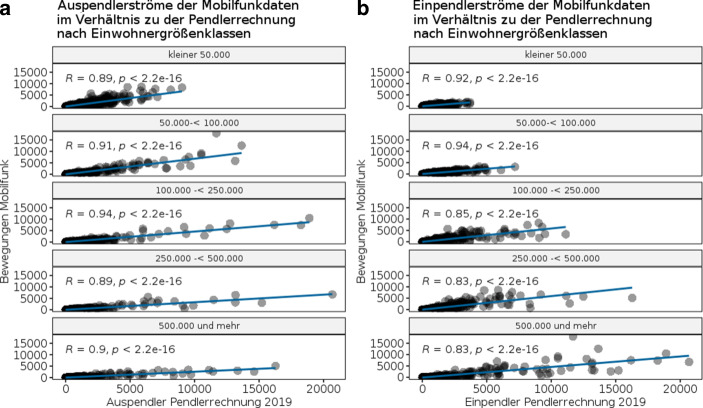

Um ergänzend zu Tab. 1 unterschiedlich strukturierte Regionen und damit mögliche regionale Unterschiede beim Vergleich der Pendlerrechnung und der Mobilfunkdaten berücksichtigen zu können, werden in Abb. 2a, b die Pearson-Korrelationskoeffizienten für die übereinstimmenden Ein- und Auspendlerströme, differenziert nach fünf Einwohnergrößenklassen, betrachtet. Die Einwohnergrößenklassen geben die Anzahl der Einwohner je Gemeinde in gleichgroßen Klassen wieder, beginnend bei Gemeinden mit weniger als 50.000 Einwohnern bis hin zu 500.000 Einwohnern aufwärts. Weiterhin wird die absolute Anzahl der Ein- und Auspendlerströme zwischen den Mobilfunkdaten 2019 und der Pendlerrechnung 2019 anhand der Einwohnergrößenklasse in einem Streudiagramm gegenübergestellt. Auf der x‑Achse sind die absoluten Zahlen der Aus- und Einpendlerströme der Pendlerrechnung hinterlegt und auf der y‑Achse die der Mobilfunkbewegungen der Tagespendler. Ebenfalls finden sich für jede Gegenüberstellung die Korrelationskoeffizienten je Einwohnergrößenklasse.

Abb. 2a, b zeigen, dass die Korrelationen über alle Einwohnergrößenklassen hinweg einen sehr starken positiven linearen Zusammenhang sowohl bei den Ein- wie auch Auspendlerströmen aufweisen und daher auf ähnliche Intensitäten der Pendlerströme hindeuten. Trotz des insgesamt sehr starken positiven linearen Zusammenhangs beider Datenquellen in allen Einwohnergrößenklassen werden die absoluten Pendlerströme in den Mobilfunkdaten bei den Ein- und Auspendlerströmen im Vergleich zur Pendlerrechnung deutlich unterschätzt. Dies wird bereits an den unterschiedlichen x‑ und y‑Achsenlängen ersichtlich.^21^ Größere Gemeinden oder Städte mit einer Einwohnerzahl ab 250.000 Einwohnern aufwärts deuten bspw. den schwächsten linearen Zusammenhang mit Korrelationskoeffizienten unter 0,9 an. In diesen Fällen ist davon auszugehen, dass die Pendlerströme in größeren bzw. einwohnerreichen Gemeinden mit den Mobilfunkdaten tendenziell unterschätzt werden. Als Beispiel für die systematische Unterschätzung wird in Tab. 2 eine Gegenüberstellung der 10 größten (absoluten) Ein- und Auspendlerströme der Stadt Köln anhand der Anzahl von Ein- und Auspendlern nach der Pendlerrechnung 2019 und den Mobilfunkdaten 2019 betrachtet sowie die prozentuale Abweichung der gelisteten Mobilfunkdatenströme zur Pendlerrechnung. Die prozentuale Abweichung der Mobilfundkatenströme richtet sich dabei nach den identischen Pendlerströmen der Pendlerrechnung. Letztere sind daher nicht zwangsläufig bzw. als Pendant in Tab. 2 aufgeführt.

**Table 2:** 

Pendlerrechnung 2019		Mobilfunkdaten 2019		
**Auspendlerströme (Wohnort Köln)**				
*Arbeitsort*	*Anzahl Auspendler*	*Arbeitsort*	*Anzahl Auspendler*	*Abweichung zur Pendlerrechnung in %*
Bonn, Stadt	16.275	Bonn, Stadt	5103	−68,65
Düsseldorf, Stadt	14.608	Leverkusen, Stadt	3292	−70,41
Leverkusen, Stadt	11.125	Düsseldorf, Stadt	2603	−82,18
Bergisch Gladbach, Stadt	7705	Bergisch Gladbach, Stadt	2513	−67,38
Hürth, Stadt	6112	Troisdorf, Stadt	1869	−56,26
Frechen, Stadt	5036	Wesseling, Stadt	1805	−23,32
Troisdorf, Stadt	4273	Hürth, Stadt	1726	−71,76
Pulheim, Stadt	3926	Brühl, Stadt	1544	−46,41
Brühl, Stadt	2881	Frechen, Stadt	1325	−73,69
Kerpen, Stadt	2818	Dormagen, Stadt	1091	−57,93
**Einpendlerströme (Arbeitsort Köln)**				
*Wohnort*	*Anzahl Einpendler*	*Wohnort*	*Anzahl Einpendler*	*Abweichung zur Pendlerrechnung in %*
Bergisch Gladbach, Stadt	18.245	Pulheim, Stadt	17.952	53,66
Leverkusen, Stadt	16.181	Hürth, Stadt	12.557	−7,98
Bonn, Stadt	15.215	Frechen, Stadt	9145	−13,05
Hürth, Stadt	13.646	Leverkusen, Stadt	7511	−53,58
Pulheim, Stadt	11.683	Bergisch Gladbach, Stadt	7365	−59,63
Frechen, Stadt	10.517	Niederkassel, Stadt	6968	20,22
Düsseldorf, Stadt	9717	Erftstadt, Stadt	4807	−24,77
Kerpen, Stadt	9681	Brühl, Stadt	4477	−29,18
Troisdorf, Stadt	8890	Bornheim, Stadt	4321	−12,69
Bergheim, Stadt	7783	Dormagen, Stadt	4057	−29,04

Dabei werden in Tab. 2 exemplarisch zwei Kernergebnisse dieses Artikels verdeutlicht, die auch in anderen Pendlerverflechtungen NRWs auf Basis der Mobilfunkdaten vorzufinden sind. Zum einen wird in den Mobilfunkdaten eine andere Reihenfolge der größten Ein- und Auspendlerströme nach und aus Köln wiedergegeben als in der amtlichen Pendlerrechnung. Zum anderen werden alle Pendlerverflechtungen mit den Mobilfunkdaten – unter anderem mit Ausnahme der Stadt Pulheim – deutlich unterschätzt, wie auch die prozentuale Abweichung zur Pendlerrechnung in Tab. 2 verdeutlicht. Pulheim ist eine an Köln angrenzende Stadt mit weniger als 50.000 Einwohnern und der ersten Einwohnergrößenklasse zugeordnet, deren Einpendlerströme um mehr als 6000 Einpendler überschätzet werden. Insgesamt wird jedoch die Anzahl der gesamten Pendlerströme in NRW mit den Mobilfunkdaten um ca. −49,79 % unterschätzt, wie die absoluten Werte der Ein- und Auspendler in den Mobilfunkdaten in Tab. 2 zeigen.

Tab. 3 listet abschließend den Anteil der Gemeinden auf, deren Pendlerverflechtungen nach der Pendlerrechnung 2019 mit den vorliegenden Mobilfunkdaten über- oder unterschätzt werden. Dies wird, wie in Abb. 2, differenziert nach Einwohnergrößenklassen sowie durch eine Unterscheidung nach Ein- und Auspendlerströmen betrachtet. Grundsätzlich wird nochmals deutlich, dass mit den Mobilfunkdaten besonders einwohnerarme Gemeinden überschätzt werden (vgl. Tab. 3). Bei ca. 41–42 % der enthaltenen Gemeinden mit weniger als 50.000 Einwohnern werden die Ein- und Auspendlerströme durch die verfügbaren Mobilfunkdaten überschätzt. D. h., die absoluten Mobilfunkdatenströme sind größer als die der Pendlerrechnung 2019, wie am Beispiel der Stadt Pulheim bereits exemplarisch abgeleitet wurde (vgl. Tab. 2). Je größer die Einwohnergrößenklasse jedoch wird, desto weniger werden die Ein- und Auspendlerströme mit den Mobilfunkdaten überschätzt und tendieren eher dazu diese zu unterschätzen. Insbesondere bei ca. 97 % der Gemeinden mit 500.000 Einwohnern und mehr werden die Einpendlerströme unterschätzt sowie bei 93 % dieser Gemeinden hinsichtlich der Auspendlerströme. Der Anteil an Gemeinden, deren Pendlerströme mit den Mobilfunkdaten identisch geschätzt werden können, ist dagegen vernachlässigbar gering. Zusammenfassend kann somit ein Stadt-Land- oder auch Stadt-Umland-Gefälle in den Mobilfunkdaten impliziert werden, bei dem die vorliegenden Mobilfunkdaten die Bewegungen in urbanen oder einwohnerreichen Gebieten deutlich unterschätzen und jene in ländlicheren oder einwohnerärmeren Gebieten tendenziell überschätzen.^22^ Von einer zeitlich schnelleren Abbildung von Pendlerströmen mittels Mobilfunkdaten als experimentelle Pendlerrechnung muss aufgrund der deutlichen Unter- bzw. Überschätzung der Pendlerverflechtungen daher abgesehen werden.

**Table 3:** 

Einwohnergrößenklasse	Schätzung der Auspendlerströme pro Gemeinde in %			Schätzung der Einpendlerströme pro Gemeinde in %		
	überschätzt	unterschätzt	identisch	überschätzt	unterschätzt	identisch
kleiner 50.000	41,30	56,22	2,47	41,81	55,54	2,65
50.000 ≤ 100.00	29,87	68,39	1,74	38,29	59,91	1,81
100.000 ≤ 50.000	20,38	78,03	1,58	14,74	84,10	1,12
250.000 ≤ 500.00	11,82	87,53	0,65	7,09	92,37	0,05
500.000 und mehr	5,83	93,20	0,97	2,23	97,35	0,04

### Kleinräumige Pendlerbewegungen in Städten – eine erweiterte Zielorts-Bestimmung

Die Abgleiche beider Datenquellen machen deutlich, dass die vorliegenden Mobilfunkdaten grundsätzlich das Potenzial haben, die Pendlerverflechtungen der amtlichen Pendlerrechnung abzubilden und zu unterstützen. Eine Erweiterungsmöglichkeit der Pendlerrechnung findet sich daher in der kleinräumigen Darstellung der Pendlerverflechtungen unterhalb der Gemeindeebene, auf der die amtliche Pendlerrechnung die Ergebnisse aktuell maximal publizieren kann. Bislang liegen keine Informationen zu innerstädtischen Verflechtungen oder besonders stark frequentierten kleinräumigen Zielorten vor. Solche räumlich differenzierten Betrachtungsmöglichkeiten liefern Informationen zur Lokalisierung oder Abgrenzung von Arbeits- und Wohngebieten und produzieren eine wertvolle Grundlage zur Infrastrukturnutzung sowie deren Planung und Ausbau. Darüber hinaus können die Pendlerverflechtungen geografisch feiner betrachtet werden.

Die zuvor durchgeführten Korrelationsanalysen unterstützen dabei eine für die kleinräumige Darstellung notwendige Bedingung, bei der sich die zielgerichteten Bewegungen in den Mobilfunkdaten sowie der Pendlerrechnung sehr stark ähneln müssen. Daher kann weiterhin angenommen werden, dass die Mobilfunkdaten, die sich auf regional größeren Gebieten mit der Pendlerrechnung stark gleichen, ebenfalls aufschlussreiche Informationen zu kleinräumigen Gebieten liefern und damit eine potenziell wertvolle Erweiterung der amtlichen Pendlerrechnung bieten können. Insbesondere soll daher analysiert werden, wohin die Berufspendler pendeln und wie stark die Zielorte frequentiert werden, wenn sich diverse Arbeitgeber in der Zielgemeinde befinden.

Da die Mobilfunkdaten den Zielort bzw. den potenziellen Arbeitsort für Städte mit mehr als 100.000 Einwohnern auf Ebene der Gitterzellen mit Gitterweiten ab 250 × 250 m bis hin zu 4 × 4 km beinhalten, wird nachfolgend die Anzahl der potenziellen Einpendler je Zielort bzw. vermeintlichen Arbeitsort der Berufspendler aufsummiert und zusammengefasst dargestellt. Da nur die Zielorte der Bewegungsströme in den Mobilfunkdaten auf Ebene der Gitterzellen zur Verfügung stehen und nicht der Wohnort bzw. die Startgemeinde, werden im Folgenden nur die Einzugsgebiete der Einpendler in den Mobilfunkdaten betrachtet. Wir betrachten somit in der kleinräumigen Zielorts-Bestimmung die frequentierten Zielorte aller Einpendler und lassen dabei die Herkunft oder auch Quelle der Bewegungen außer Acht. Hierbei fließen dementsprechend nur die mit der Pendlerrechnung übereinstimmenden Mobilfunkbewegungen ein.

Weiterhin wurde im vorherigen Abschnitt bereits aufgezeigt, dass die Mobilfunkdaten die Pendlerströme in einwohnerreicheren Gemeinden deutlich unterschätzen. Daher fallen auch die absoluten Zahlen der Pendlerbewegungen in den kleinräumigen Zielorten tendenziell zu gering aus, weshalb der Fokus vorrangig auf die Lokalisierung der Zielorte und die regional bzw. urban stärksten Einzugsgebiete der Berufspendler gerichtet wird.

**Figure 3:**
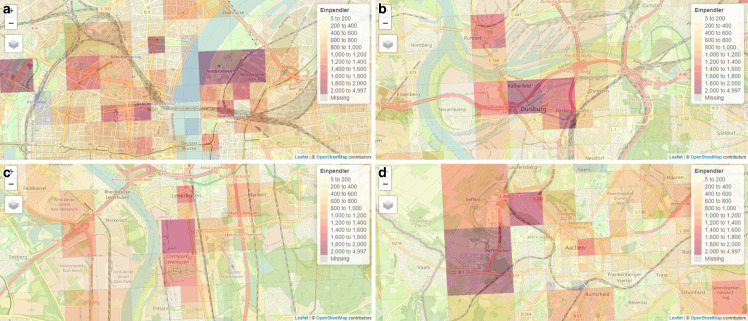

Zur Veranschaulichung der Kernergebnisse greifen wir hierzu auf Beispiele in Abb. 3 zurück, die aus der zugehörigen interaktiven Karte (Onlinematerial 1) entnommen wurden.^23^ Die Darstellung visualisiert die Einzugsgebiete der ermittelten Einpendler auf Ebene der unterschiedlich großen Gitterzellen. Da sich die Größe der Gitterzellen an dem Mobilfunknetz des Anbieters orientiert und damit auch an der Dichte der Mobilfunknutzenden im Untersuchungsgebiet, sind Stadtzentren bzw. Innenstädte entsprechend mit kleinen Gitterzellen ausgelegt und weniger dicht besiedelte städtische Randbezirke mit größeren Gitterweiten. Damit wird es bereits möglich die Stadtzentren anhand der Gitterweiten zu lokalisieren. Um jedoch auf die durch die Berufspendler stärker frequentierten Einzugs- bzw. Arbeitsgebiete schließen zu können, wird die Anzahl der Einpendler je Gitterzelle farblich hervorgehoben. Je dunkler die Gitterzelle schattiert ist,^24^ desto höher ist die Anzahl der einpendelnden Berufspendler aus den Mobilfunkdaten in der entsprechenden Gitterzelle. Für die Verortung der frequentierten Gitterzellen und Interpretation derselben sind zusätzliche Geodaten von OpenStreetMap^25^ in den Karten hinterlegt.

**Figure 4:**
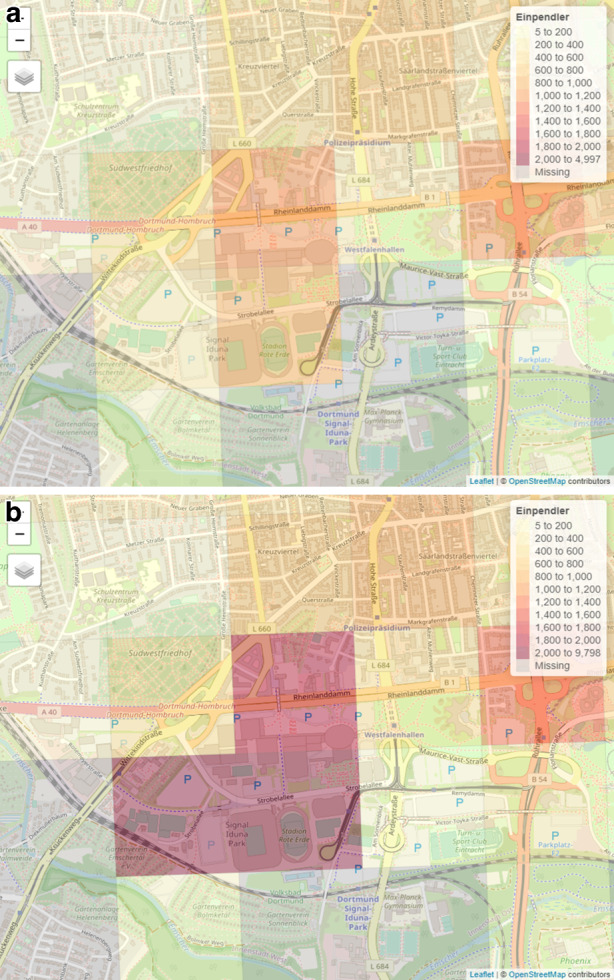

Die Ermittlung und Visualisierung der kleinräumigen Mobilfunkbewegungen der potenziellen Berufseinpendler offenbaren vier charakteristische Zielorte, die sich teilweise klar den Berufspendlern zuordnen lassen sowie andere, bei denen es sich vermutlich um keine Arbeitsstätte handelt. Die Beispiele sind in Abb. 3 hinterlegt.

Einen großen Einfluss auf die Zielorte der kleinräumigen Bewegungsverflechtungen in den Mobilfunkdaten haben Gewerbe- und Industriestandorte. Unter anderem bieten Chemieparks^26^, bspw. in Leverkusen oder Krefeld, zahlreiche Arbeitsplatzangebote in den Regionen, die auch in den Zielorten der Mobilfunkdaten stark frequentiert werden (siehe Abb. 3c). Zudem bilden Gewerbegebiete, wie die Duisburg-Ruhrorter Häfen, Kernpfeiler der Arbeitsmarktregion in Städten wie Duisburg (siehe Abb. 3b). Der Mediapark in Köln, ein für Medienunternehmen konzipierter Gewerbepark (siehe Abb. 3a), oder das Vallourec Deutschland, ein Walzwerk in Düsseldorf-Rath, sind genauso stark frequentierte Einzugsgebiete der Berufspendler, um nur einige Beispiele zu nennen.

Neben den offensichtlichen Produktions- oder Gewerbestätten fallen Stadtzentren, Altstädte oder Innenstädte sowie im Regelfall der dazugehörige Hautbahnhof, wie exemplarisch in Abb. 3a, b dargestellt, besonders stark auf. Sie stechen in jeder Stadt in NRW mit einer hohen Zahl an Einpendlern hervor. Stadtzentren bieten zahlreiche Arbeitsmöglichkeiten in diversen Dienstleistungen, in administrativen Tätigkeiten in der kommunalen Verwaltung o. ä., die mitunter historisch bedingt aus der Altstadt bzw. Innenstadt größerer Städte entstanden sind. Natürlich ist auch hier hervorzuheben, dass nicht ausgeschlossen werden kann, dass sich neben den gewünschten Berufspendlern auch andere freizeitbedingte mobile Bewegungen, wie bspw. die der Einkaufstouristen, in den ausgewählten Daten befinden. Durch die Filtersetzung, dass ein Eintritt der mobilen Aktivitäten in den Zielort vor 9 Uhr erfolgen muss, kann jedoch angenommen werden, dass ein Großteil dieser unerwünschten Bewegungsverflechtungen rausgefiltert wurde.

Darüber hinaus bilden Messegelände einen weiteren Zielort potenzieller Berufspendler in den Mobilfunkdaten ab. Exemplarisch hierzu treten die Gitterzellen in Abb. 3a besonders stark hervor, die das Messegelände bei Köln/Messe Deutz oder die Messe Düsseldorf beinhalten. Auch beim Messebesuch muss wieder zwischen Arbeitskräften und Messebesuchern ohne Arbeitsauftrag unterschieden werden.

Den dritten charakteristischen Zielort stellen Kliniken, unter anderem auch Universitätskliniken, dar, die eine große Anzahl von Arbeitsplätzen stellen. Bei den Kliniken treten gleichsam dieselben Herausforderungen in den Mobilfunkdaten auf wie in der Messewirtschaft oder in den Innenstädten. Zunächst können wir nicht sagen, ob es sich ausschließlich um Arbeitskräfte oder auch um Besuchende in den Daten handelt, die vor 9 Uhr eine Einrichtung bzw. ein Gelände aufsuchen. Zudem stellen Kliniken klassische Schichtbetriebe dar. Das bedeutet, dass mit dem 9 Uhr Filter in den Mobilfunkdaten im Sinne der Schichtarbeit nur die Arbeitskräfte der Frühschicht erfasst werden können. Die Spät- und Nachtschicht entfällt hiermit komplett, so dass davon auszugehen ist, dass die absolute Anzahl der Berufseinpendler in diesen Zellen unterschätzt wird. Zudem stellen unter anderem auch Universitätskliniken durch die Studierenden vor Ort eine weitere exemplarische Herausforderung in der zielorientieren Aufbereitung der Mobilfunkdaten dar, welche nachfolgend anhand des letzten charakteristischen Zielortes erörtert wird.

Bildungseinrichtungen wie allgemeinbildende Schulen oder Universitäten sind in den Mobilfunkdaten besonders stark frequentiert, wie bspw. die Rheinisch-Westfälische Technische Hochschule (RWTH) Aachen (siehe Abb. 3d) oder die Ruhr-Universität Bochum. Diese treten unter anderem stärker hervor, da ihre Fakultäten durch den Status einer Campus-Universität zentral an einem Ort vorzufinden sind und nicht – wie teilweise bei anderen Hochschulen – diffuser in der Stadt verteilt sind. Bei den Bildungseinrichtungen ergibt sich nun das Problem, dass offensichtlich Studierende sowie Schülerinnen und Schüler in den Daten enthalten sind. Laut Definition gehören sie nicht den Berufspendlern an, sondern werden separat als Bildungspendler ausgewiesen. Durch den Eintritt der mobilen Aktivitäten in den Zielort vor 9 Uhr und bspw. dem Beginn der ersten Unterrichtsstunde um 8 Uhr provozieren wir einen deutlich stärkeren Einbezug von Bildungspendlern mit den hier verwendeten Filterkriterien in den Mobilfunkdaten. Auch bei den Universitätskliniken ist es problematisch die Bildungspendler der angegliederten medizinischen Fakultät der Universität von den Berufspendlern des Klinikums zu unterscheiden. Das hängt mitunter auch mit den unterschiedlichen Gitterweiten der Gitterzellen zusammen, die keine räumlich scharfe Trennung ermöglichen. Insgesamt bedeutet das, dass die gefilterten Pendlerverflechtungen in den Mobilfunkdaten neben den potenziellen Berufseinpendlern auch die Bildungspendler beinhalten. Da die amtliche Pendlerrechnung keine Bildungspendler aufgrund von Dateninkonsistenzen ausgibt, wäre die Ermittlung der Bewegungsverflechtungen der Bildungspendler eine zusätzliche Erweiterungsmöglichkeit der Pendlerrechnung, die in weiterer Forschungsarbeit mit den Mobilfunkdaten aufgegriffen werden könnte.

Was alle charakteristischen Zielorte letztlich gemeinsam haben, ist die sehr gute Erreichbarkeit der kleinräumigen Zielorte durch das Straßen- und Schienennetz in NRW. Jede dunkel schattierte Gitterzelle enthält eine Anbindung an eine Autobahn, Bundesstraße oder einen Bahnhof oder liegt bereits in der sehr gut angebundenen Innenstadt.^27^ Eine gute räumliche Erreichbarkeit von Arbeitsplätzen bedingt somit maßgeblich die Bildung oder Verlagerung von Arbeitsmärkten. Weiterhin wird an den Beispielen in Abb. 3 deutlich, dass innerhalb der Städte durchaus sehr große innerstädtische Unterschiede hinsichtlich ihrer Frequentierung bestehen können und damit auch die Infrastruktur einer Stadt unterschiedlich stark belastet wird.

Als letztes Beispiel wird eine mögliche Konsequenz betrachtet, die entsteht, sofern die Datengrundlage zu viele nicht definierbare Bewegungsverflechtungen enthält. Damit greifen wir noch einmal die in Abschn. 3.1 geführte Diskussion hinsichtlich der für diesen Artikel passenden Personenkategorien auf.

Abb. 4 stellt das bekannte Fußballstadion in Dortmund, den Signal Iduna Park, sowie die daran angrenzenden Westfalenhallen dar. Der Signal Iduna Park bietet ca. 81.000 Plätze für Zuschauende von Fußballspielen. Die benachbarten Westfalenhallen sind ein Messe‑, Kongress- und Veranstaltungszentrum und haben eine Kapazität von 15.400 Plätzen. Beides zusammengenommen stellen sie daher besonders Zielorte für touristische bzw. freizeitliche Aktivitäten und weniger für Berufspendler dar, wie auch Abb. 4 verdeutlicht. In Abb. 4a sind die Summen der Einpendler unter Einbezug der Tagespendler abgebildet und in Abb. 4b die Summen der Einpendler unter Einbezug aller Personenkategorien, einschließlich der Tagestouristen. Den Spitzenwert der Skala mit einem Besucherniveau von durchschnittlich 9798^28^ Einpendlern ist am Signal Iduna Park durch den Einbezug aller in den Mobilfunkdaten befindlichen Bewegungsverflechtungen erreicht, während es unter Einbezug der Tagespendler nur 978 gezählte Einpendler sind. Findet ein Fußballspiel oder bspw. ein Konzert an einem in den Daten enthaltenen Werktag statt, wird durch die sehr hohe Besucheranzahl das durchschnittliche Besucherniveau in den jeweiligen Gitterzellen, wie in Abb. 4b, entsprechend angehoben.

Dieses Phänomen wird bei allen Bundesligastadien in NRW sichtbar und liefert insofern gegensätzliche Aussagen zu der Zielsetzung dieser Arbeit. Damit ist ein Einbezug aller Daten für die Erweiterung der amtlichen Pendlerrechnung bewiesenermaßen nicht geeignet. Dieses Beispiel zeigt eindrücklich, wie essenziell eine nicht wohldurchdachte Datengrundlage die Ergebnisse hinsichtlich der Messung von Berufspendlern beeinflussen oder verzerren kann. Ähnliche aber weit weniger drastische Effekte werden bei Naherholungsgebieten wie Naturparks, Halden oder großen Einkaufzentren, wie bspw. das CentrO in Oberhausen, sichtbar.

### Zusammenhänge zwischen Berufspendlern nach Beschäftigungsumfang und Mobilfunkdaten

Die amtliche Pendlerrechnung gibt neben der Pendlerart auch Informationen zum Beschäftigungsumfang der Berufspendler wieder. Der Beschäftigungsumfang ermöglicht Angaben zum Umfang der erbrachten Arbeitsleistung der Beschäftigten. Diese werden nachfolgend in Teilzeit- und Vollzeitbeschäftigung sowie die zusammengefasste Beschäftigung unterschieden. Unter Teilzeitbeschäftigung werden nach § 2 Teilzeit- und Befristungsgesetz (TzBfG) Arbeitnehmende verstanden, deren regelmäßige Wochenarbeitszeit kürzer ist als die der vergleichbaren vollzeitbeschäftigten Arbeitnehmenden. Die in Teilzeit zu leistende Arbeitszeit kann jedoch sehr unterschiedlich gestaltet sein und sehr flexibel eingeteilt werden und zählt theoretisch bereits bei weniger als 40 Wochenarbeitsstunden als solche. Daher ergibt sich die Fragestellung, inwieweit die vorliegenden Mobilfunkdaten diese beiden allgemeinen Arbeitszeitmodelle beinhalten und abbilden können. Insbesondere die eingesetzte zweistündige Verweildauer der mobilen Aktivitäten in einem Untersuchungsgebiet könnte zu kurz erscheinen, um nur Berufspendler aus den Mobilfunkdaten zu extrahieren. Daher betrachten wir im Folgenden weitere Zusammenhänge der Mobilfunkdaten mit der Pendlerrechnung, ohne dabei auf die konkreten Pendlerverflechtungen einzugehen. Stattdessen wird die Verweildauer in den Mobilfunkdaten mit dem Beschäftigungsumfang der Berufspendler aus der Pendlerrechnung in Zusammenhang gebracht. Anhand dessen soll überprüft werden, inwieweit die gegebene Verweildauer geeignet ist, um die Berufspendler insgesamt sowie unterteilt nach Beschäftigungsumfang darzustellen.

Im Vergleich zu den Pendlerverflechtungen werden die Berufspendler nach Beschäftigungsumfang in der amtlichen Pendlerrechnung nicht in Bewegungen bzw. Verflechtungen ausgedrückt, sondern als Jahresdurchschnitt pro Gemeinde angegeben. Selbige Auflösung findet sich auch in den Mobilfunkdaten wieder. Es werden die Tagessummen der mobilen Aktivitäten im Untersuchungsgebiet und damit dem potenziellen Arbeitsort wiedergegeben. Zusätzlich liegen in den Mobilfunkdaten die durchschnittlichen Verweilzeiten aller gruppierten mobilen Aktivitäten in einem Halbstundentakt, von einer halben Stunde bis hin zu 23 h Verweilzeit, im Untersuchungsgebiet vor. Diese Information bietet uns die Möglichkeit, die Verweildauer in den Mobilfunkdaten in Zusammenhang mit dem Beschäftigungsumfang der Berufspendler zu setzen und mögliche Gemeinsamkeiten beider Datenquellen sowie Alternativen aufzuzeigen.

Für die Ermittlung möglicher Zusammenhänge werden erneut die Korrelationen zwischen dem Beschäftigungsumfang und der Pendlerart aus der amtlichen Pendlerrechnung zu den Tagessummen in den Mobilfunkdaten in Abhängigkeit von der halbstündigen Verweildauer innerhalb der Mobilfunkdaten berechnet. Damit soll zum einen analysiert werden, inwieweit die zweistündige Verweildauer in den Daten gerechtfertigt ist oder ob eine andere Verweilzeit in den Mobilfunkdaten den Beschäftigungsumfang besser abbilden kann.

**Figure 5:**
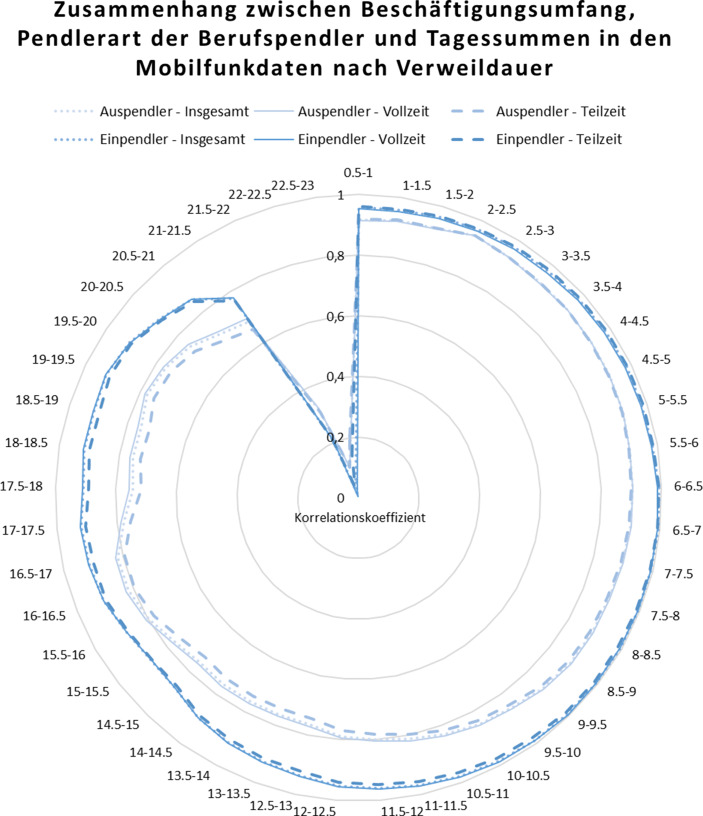

Abb. 5 stellt die Korrelationen zwischen Beschäftigungsumfang und Pendlerart aus der amtlichen Pendlerrechnung zu den Tagessummen aus den Mobilfunkdaten in einem Netzdiagramm dar. Die Besonderheit in den berechneten Korrelationen liegt darin, dass diese in Abhängigkeit von der halbstündigen Verweildauer in den Mobilfunkdaten gebildet wurden und damit pro Verweildauer ein Korrelationswert ausgegeben wird. Die gepunkteten oder durchzogenen Datenreihen geben die Pearson-Korrelationskoeffizienten der Aus- und Einpendler nach Vollzeitbeschäftigung, Teilzeitbeschäftigung sowie insgesamt wieder. Das Hauptgitternetz beinhaltet die Korrelationskoeffizienten in Bezug zu den halbstündigen Verweilzeiten als Rubrikenachsenbeschriftung, beginnend bei 0,5–1 h bis hin zu 22,5–23 h Verweildauer. Hierbei gilt, dass je weiter außen am Rand des Hauptgitternetzes die Linien der sechs Datenreihen verlaufen, desto höher ist die Korrelation zwischen den eingeordneten Pendlern der Pendlerrechnung und den Mobilfunkdaten. Anhand der Kreisverläufe können sodann Rückschlüsse auf die Informationsgüte der einzelnen Verweilzeiten geschlossen werden.

Zunächst kann anhand von Abb. 5 festgestellt werden, dass grundsätzlich zwischen der Anzahl der Pendler nach Beschäftigungsumfang sowie der Pendlerart aus der Pendlerrechnung und den Tagessummen der Mobilfunkdaten eine sehr hohe Korrelation besteht. Insgesamt werden jedoch Unterschiede bei der Differenzierung der Korrelationen nach der Pendlerart offensichtlich, genauer zwischen Ein- und Auspendlern.

Die vorliegenden Mobilfunkdaten sind mit einer Verweildauer von zwei Stunden aufbereitet worden. Aus Abb. 5 wird nun ersichtlich, dass sich diese Verweildauer für die ausschließliche Abbildung der vollzeit- oder teilzeitbeschäftigten Einpendler weniger eignet. Stattdessen offenbaren die Korrelationskoeffizienten marginale Unterschiede in der Verweildauer nach Teilzeit- und Vollzeitbeschäftigung, die insbesondere bei längeren Verweildauern sichtbar werden. Bei den Einpendlern würde die höchste Korrelation (zwischen Einpendlern nach Beschäftigungsumfang aus der Pendlerrechnung und den Tagessummen der Mobilfunkdaten) und damit die optimale Verweilzeit zur Abbildung der Teilzeitbeschäftigten (*Einpendler – Teilzeit*) in den Mobilfunkdaten bei einer Verweildauer von 6,5–7 h liegen und bei den Vollzeitbeschäftigten (*Einpendler – Vollzeit*) bei 8,5–9 h (siehe Rubrikenachsenbeschriftung in Abb. 5).

Im Gegensatz dazu weisen die Auspendler einen anderen Trend auf. Die Korrelationen der Auspendler nach dem Beschäftigungsumfang sind insgesamt niedriger als die der Einpendler, auch wenn sie für sich genommen mit Korrelationskoeffizienten um 0,8 ebenfalls sehr hoch sind (siehe Abb. 5). Hier zeigt sich jedoch, dass eine niedrige Verweildauer von 2– 2,5 h die höchste Korrelation zwischen Beschäftigungsumfang der Auspendler zu den Mobilfunkdaten verursacht. Das ist dahingehend nicht verwunderlich, da die Tagessummen in den Mobilfunkdaten die Hauptaktivitäten am Tag je Untersuchungsgebiet und damit die durchschnittlichen Aktivitäten am potenziellen Arbeitsort wiedergeben. Damit werden nicht die durchschnittlichen Aktivitäten am potenziellen Wohnort angesprochen, was wiederum für die Ermittlung der Auspendler erforderlich wäre. D. h., die Tagessummen in den Mobilfunkdaten sind theoretisch nicht geeignet die Auspendler für die Gemeinden wiederzugeben. Insgesamt lässt sich die Fragestellung in Bezug auf die Zusammenhänge zwischen Beschäftigungsumgang und Pendlerart für die Einpendler beantworten, wobei die Ergebnisse für die Auspendler mit Vorsicht betrachtet werden müssen.

## Diskussion einer alternativen Mobilfunkdatenaufbereitung

### Einflüsse auf die Pendlerbewegungen in den Mobilfunkdaten

Die vorangegangenen Betrachtungen aus Abschn. 3 zu den Vergleichen mit der Pendlerrechnung, zur kleinräumigen Zielorts-Bestimmung sowie den Zusammenhängen mit dem Beschäftigungsumfang geben Grund zur Annahme, dass die Mobilfunkdaten diversen Einflüssen unterliegen, welche die Ergebnisse maßgeblich beeinflussen. Neben offensichtlichen methodischen Aspekten, wie die Verteilung und Extrapolation der mobilen Aktivitäten durch den Datenanbieter, auf Letzteres wird in Abschn. 4.2 kurz eingegangen,^29^ betrachten wir nachfolgend Einflüsse weiterer Variablen auf die Mobilfunkdaten.

Wie zuletzt bereits diskutiert, hat die in den Mobilfunkdaten eingebrachte Verweildauer einen beträchtlichen Einfluss auf die Verortung und die Quantität der mobilen (stationären) Aktivitäten in Form von Tagessummen. Im Gegensatz zu Abschn. 3.3 werden folgend erneut die halbstündigen Verweildauern, diesmal jedoch in Abhängigkeit von den Pendlerverflechtungen, betrachtet. Die mit der Pendlerrechnung übereinstimmenden Pendlerverflechtungen in den Mobilfunkdaten werden dabei anhand der Verweildauer korreliert. Dasselbe Verfahren wird erstmals für die durchschnittliche Distanz bzw. Entfernung zwischen potenziellem Wohn- und Arbeitsort durchgeführt.

**Figure 6:**
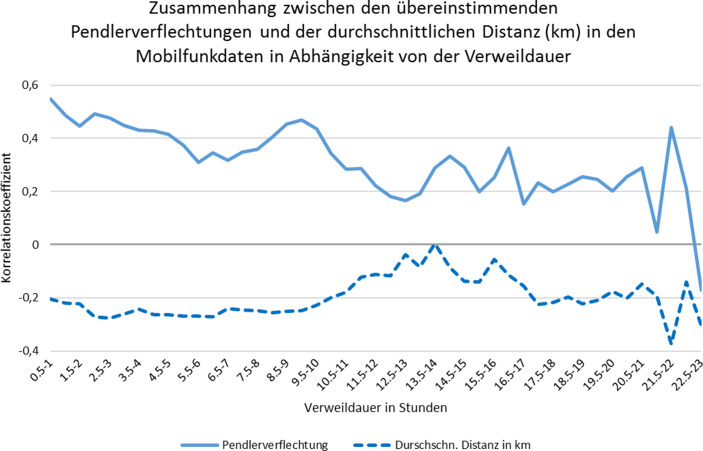

Die durchschnittliche Distanz dient als Maßstab, um unplausible Pendlerwege herauszufiltern. Daher wird davon ausgegangen, dass die zurückgelegte Distanz ebenfalls von der Verweildauer abhängen könnte, da annahmegemäß Berufspendler länger an ihrem Zielort verweilen. Möglicherweise hat auch eine größere Entfernung zwischen Wohn- und Arbeitsort einen längeren Aufenthalt am Zielort zur Folge. Abb. 6 stellt daher die Zusammenhänge zwischen den übereinstimmenden Pendlerverflechtungen beider Datenquellen sowie die Zusammenhänge zwischen Pendlerverflechtungen aus den Mobilfunkdaten und der durchschnittlich hinterlegten Distanz vom Wohn- zum Arbeitsort, jeweils in Abhängigkeit von der halbstündigen Verweildauer, in einem Liniendiagramm dar.

Zunächst kann festgestellt werden, dass die übereinstimmenden Pendlerverflechtungen aus beiden Datenquellen grundsätzlich mittel bis stark positiv mit der Verweildauer korrelieren. Der Trend in Abb. 6 deutet jedoch darauf hin, dass die Korrelation sinkt, je höher die Verweildauer wird. Das bedeutet, je länger die Verweildauer am Zielort andauert, desto weniger Einfluss hat diese auf die potenziellen Pendlerverflechtungen in den Mobilfunkdaten und desto schlechter wird die amtliche Pendlerrechnung mit den vorliegenden Mobilfunkbewegungen abgebildet.

Weiterhin kann aufgedeckt werden, dass die durchschnittliche Distanz eher eine negativ schwache bis mittlere Korrelation mit der zielgerichteten Anzahl der Mobilfunkbewegungen in Abhängigkeit von der Verweildauer aufweist. Das bedeutet, dass theoretisch die durchschnittlich zurücklegte Distanz bei steigenden Pendlerverflechtungen in den Mobilfunkdaten sinkt. Ein klarer Trend ergibt sich, wie bei der Korrelation zwischen der übereinstimmenden Pendlerverflechtung und Verweildauer, jedoch nicht, da die Korrelationskoeffizienten bei steigender Verweildauer von bis zu 14 h sogar gar keine Zusammenhänge zwischen Distanz und Mobilfunkbewegungen mehr aufweisen. Der Einfluss der Verweildauer erscheint in diesem Fall eher diffus zu sein.

Dennoch ist anhand von Abb. 6 ein Einfluss der Distanz auf die Pendlerverflechtungen in den Mobilfunkdaten erkennbar, wenn auch nicht oder nur mäßig in Abhängigkeit von der Verweildauer. Daher fokussieren wir uns in einem zweiten Schritt auf das reine Verhältnis von Distanz zu den Pendlerverflechtungen der Mobilfunkdaten.

**Figure 7:**
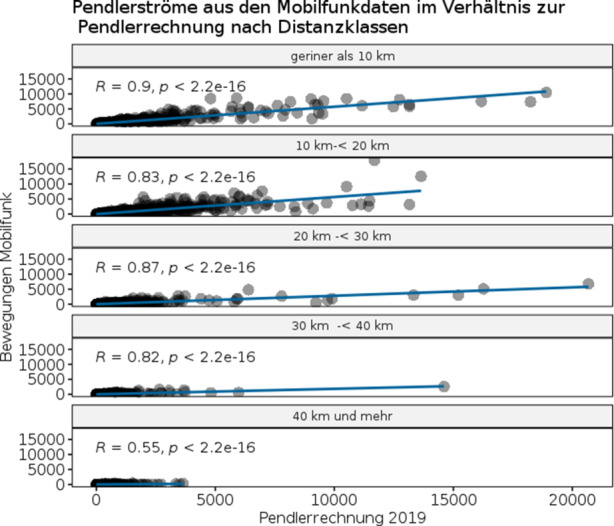

Für eine übersichtliche Darstellung werden fünf Distanzklassen beginnend mit weniger als 10 km Luftlinienentfernung bis hin zu 40 km und mehr gebildet, wobei die maximale Entfernung vom Wohn- zum Arbeitsort, entsprechend der Methodik der Pendlerrechnung, maximal 80 km betragen darf. Die in die Distanzklassen unterteilten Zusammenhänge zwischen den übereinstimmenden Pendlerverflechtungen aus den Mobilfunkdaten und der Pendlerrechnung werden in Abb. 7 wiedergegeben. Wie auch in Abb. 2 sind die absoluten Zahlen der Pendlerströme der Mobilfunkdaten 2019 auf der y‑Achse und die der Pendlerrechnung 2019 auf der x‑Achse abgetragen. Ebenfalls finden sich für jede Gegenüberstellung die Korrelationskoeffizienten je Distanzklasse in der Darstellung.

Ergänzend zur ersten Annäherung des Einflusses der Distanz auf die Mobilfunkdaten (vgl. Abb. 6) ergibt sich aus Abb. 7 weiterhin, dass zwischen der Pendlerrechnung und den Mobilfunkdaten in den ersten vier Distanzklassen bis zu 40 km, hier definiert und nachfolgend bezeichnet als Kurzdistanzwege, ein sehr starker positiver Zusammenhang besteht. Dieser ist in der kleinsten Distanzklasse am größten mit einem Pearson-Korrelationskoeffizient von 0,9 und bei steigender Distanz tendenziell abnehmend und folglich in der letzten bzw. größten Distanzklasse (40 km und mehr) mit einem Korrelationskoeffizienten von nur noch 0,55 am geringsten.

Da die Zahlen der Pendlerrechnung von den Filterkriterien oder Distanzmaßen der Mobilfunkdaten nicht beeinflusst werden, ergeben sich die Effekte nur aus den Mobilfunkdaten. Daraus lässt sich ableiten, dass mit steigender Distanz die Bewegungsverflechtungen in den Mobilfunkdaten mit denen der Pendlerrechnung weniger übereinstimmen und die Distanz somit, wie anhand der Abhängigkeit von der Verweildauer bereits angedeutet, einen negativen Einfluss auf die Mobilfunkdaten zu haben scheint.

Für das Erweiterungspotenzial der Pendlerrechnung durch Mobilfunkdaten können wir daraus zudem schlussfolgern, dass eine Erweiterung mit Bewegungsverflechtungen und einer zurückgelegten Distanz von bis zu 40 km (Kurzdistanzwege) aus den Mobilfunkdaten am geeignetsten sind, da diese die Pendlerverflechtungen aus der Pendlerrechnung am wahrscheinlichsten wiedergeben können. Bei solchen Kurzdistanzwegen kann auf Pendlerverflechtungen geschlossen werden, die von angrenzenden oder räumlich nahe gelegenen Gemeinden zur Zielgemeinde führen. Besonders interessant wird diese Erkenntnis für eine Erweiterung der Pendlerrechnung mit der kleinräumigen Zielorts-Bestimmung, kleinräumigen Pendlerverflechtungen oder auch die Angabe innergemeindlicher Berufspendler in Form einer *experimentellen kleinräumigen Pendlerrechnung*. Nehmen wir die Stadt Köln als Beispiel, so würden wir es schaffen mit den Kurzdistanzwegen allein in der Stadt zu verleiben und damit räumlich tiefere Betrachtungen der innergemeindlichen Pendler durchführen zu können, sofern geeignete Daten vorlägen. Dennoch bleibt auch in diesem Fall das Problem bestehen, dass mit den Mobilfunkdaten die Anzahl der mit der Pendlerrechnung übereinstimmenden Pendlerströme in allen Distanzklassen deutlich unterschätzt wird (siehe x‑y-Achse in Abb. 7).

Durch die Aufbereitungsart der Mobilfunkdaten, wie bspw. die Anwendung diverser Zeit- und Personenfilter, ist ein spürbarer Anteil an Bewegungsverflechtungen aus den Daten verloren gegangen. Weiterhin wurde bereits beleuchtet, dass die Verweildauer oder die Distanz ebenfalls einen Einfluss auf die in den Mobilfunkdaten befindlichen Bewegungsverflechtungen haben. In einem letzten Schritt wollen wir diese sowie weitere mögliche Einflüsse auf die reinen gezählten Mobilfunkaktivitäten bzw. -verflechtungen in einem Regressionsmodell quantifizieren.

Für die vorliegenden Mobilfunkdaten eignen sich sogenannte Zählmodelle zur Modellierung von Zähldaten, wobei die Mobilfunkdaten die Anzahl der täglichen, zielgerichteten Pendlerverflechtungen der einbezogenen Werktage eines dreimonatigen Zeitraums in 2019 wiedergeben. Für die Modellierung von Zähldaten wird in der Regel die Poisson Regression herangezogen, welche jedoch anfällig ist bei Verletzung der Annahme, dass der Erwartungswert und die Varianz der Verteilung gleich λ sind. Dabei beschreibt λ die mittlere Anzahl der zu erwartenden Ereignisse. Verletzen wir diese Annahme, kann dies, wie im vorliegenden Fall, zu einer Überdispersion führen. Eine Überdispersion liegt vor, wenn die Varianz größer als der Erwartungswert ist.^30^ Sie kann zur Über- oder Unterschätzung der Standardfehler der Regressionskoeffizienten führen und dadurch zu einer fehlerhaften Einschätzung der Signifikanz derselben (Hilbe 2011, Kap. 7). Um der vorliegenden Überdispersion entgegenzuwirken,^31^ findet das dafür empfohlene negative Binomiale Regressionsmodell Verwendung.^32^ Dieses nimmt die unbeobachtete Heterogenität, durch welche die Überdispersion entsteht, durch einen zusätzlichen Parameter auf (für Details siehe Hilbe 2011, Kap. 8).

In der durchgeführten negativen Binomialen Regression stellen die gezählten Mobilfunkbewegungen in Abhängigkeit aller möglichen enthaltenen Einflussfaktoren in den Mobilfunkdaten die zu interessierende Größe dar. Als zu erklärende Variablen fließen die bereits diskutierte Verweildauer (*verweildauer*), die Distanz vom Wohn- zum Arbeitsort (*dist_mean*) sowie die bekannten Einwohnergrößenklassen als kategoriale Variable (*class2019…*) im Vergleich zum Referenzwert der ersten Einwohnergrößenklasse mit weniger als 50.000 Einwohnern ein. Des Weiteren sind die möglichen Auswirkungen des Zielortes auf die Anzahl der Mobilfunkdaten von Interesse, insbesondere ob Gitterzellen in der Gemeinde bzw. Stadt enthalten sind (*TypStadt mit MTC*) oder ob eine Stadt ohne Gitterzellen vorliegt (*TypStadt*) im Vergleich zum Referenzwert der Gemeinde mit unter 100.000 Einwohnern. Damit soll ermittelt werden, ob einwohnerreichere Gemeinden oder Städte mit mehr Bewegungsverflechtungen einhergehen.

Trotz der geringen Anzahl an Kovariaten wurde eine Modellselektion mittels einer schrittweisen Variablenselektion unter Verwendung des Akaike-Informationskriteriums durchgeführt, wobei die Auswahl des Modells unberührt blieb. Weiterhin deutet die Devianz des Modells auf einen guten Modellfit im Vergleich zu einem alternativen Poisson Modell hin.^33^

Die resultierenden Koeffizientenschätzer des negativen Binomialen Modells sind in Tab. 4 dargestellt. Für die Interpretation der Einflüsse bzw. der geschätzten Effekte der Koeffizientenschätzer ($\hat{\beta }$) auf die gezählten Mobilfunkbewegungen werden ergänzend die Exponenten der Schätzer berechnet ($\exp \left(\widehat{\beta}\right)$). Negative oder positive Auswirkungen gehen aus den Vorzeichen der Koeffizientenschätzer hervor. Die numerischen Effekte entsprechen den berechneten Exponenten unter der Voraussetzung, dass alle anderen Koeffizienten konstant gehalten werden.

**Table 4:** 

Variable	$\hat{\beta }$	$\exp \left(\widehat{\beta}\right)$	Std. Fehler	z‑Wert	PR(> |z|)
Intercept	2,824	16,846	0,006	511,76	**0,000**
verweildauer	−0,012	0,988	0,0002	−60,37	**0,000**
TypStadt	0,368	1,445	0,005	76,48	**0,000**
TypStadt mit MTC	0,640	1,897	0,007	98,11	**0,000**
class201950.000–<100.000	0,136	1,146	0,005	27,24	**0,000**
class2019100.000–<250.000	0,123	1,130	0,008	16,00	**0,000**
class2019250.000–<500.000	0,176	1,192	0,010	18,41	**0,000**
class2019500.000 und mehr	0,191	1,210	0,012	15,84	**0,000**
dist_mean	−0,024	0,976	0,0002	−125,20	**0,000**

Die Koeffizientenschätzer in Tab. 4 haben insgesamt einen signifikanten Einfluss auf die gezählten Mobilfunkdaten (siehe hierzu PR(> |z|)) und demnach einen Einfluss auf die Anzahl der mobilen Aktivitäten. Betrachten wir die Koeffizientenschätzer einzeln, bestätigen sie mitunter vorherige Erkenntnisse wie die negativen Einflüsse der Verweildauer und der Distanz auf die Anzahl an mobilen Bewegungen. Die Anzahl der Mobilfunkverflechtungen sinkt erwartungsgemäß um den Faktor 0,988, wenn die Verweildauer um eine halbe Stunde steigt und die übrigen Variablen im Modell konstant gehalten werden. Steigt die durchschnittlich zurückgelegte Distanz um einen Kilometer, so sinkt die Anzahl der Mobilfunkverflechtungen um den Faktor 0,976. Diese beiden negativen Effekte haben, im Vergleich zu den anderen Koeffizientenschätzer, den geringsten zu erwartenden Einfluss auf die Mobilfunkdaten.

Anders verhält es sich beim Zielort und der Einwohnergrößenklasse. Da diese beiden kategorialen Variablen mehrere Klassen oder Kategorien enthalten, wird im Folgenden jeweils die erste Kategorie beider Variablen als Referenzwert genommen, woran sich die Effekte der aufgelisteten Kategorien in Tab. 4 messen. Dabei stellen wir fest, dass Untersuchungsgebiete bzw. Zielorte mit weiterer Unterteilung in Gitterzellen erwartungsgemäß knapp doppelt so viele Mobilfunkverflechtungen aufweisen wie Gemeinden mit unter 100.000 Einwohnern, wobei die übrigen Variablen konstant gehalten werden (siehe Tab. 4*TypStadt mit MTC* mit Faktor 1,9). Damit üben die Untersuchungsgebiete den größten Einfluss auf die gezählten mobilen Aktivitäten aus. Diese Erkenntnisse werden auch in den Effekten der vier gelisteten Einwohnergrößenklassen gefunden. Je größer die Einwohnergrößenklasse im Vergleich zu Gemeinden mit weniger als 50.000 Einwohnern ist, desto größer wird der Faktor der jeweiligen Klasse, um den die Anzahl der Mobilfunkverflechtungen erwartungsgemäß steigt. Auch hier gilt die Schlussfolgerung nur, wenn alle anderen Variablen im Modell konstant gehalten werden.

Zusammenfassend kann daraus abgeleitet werden, dass mehr mobile Bewegungsverflechtungen in einwohnerreichen Regionen provoziert werden und diese weiterhin mit einer kurzen zurückgelegten Distanz sowie kurzer Verweildauer einhergehen. Die letzte Aussage wirkt möglicherweise widersprüchlich zur Schlussfolgerung, dass längere Verweildauern geeigneter sind, um Berufspendler abzubilden. Hier muss daher berücksichtigt werden, dass in Tab. 4 nur die Einflüsse auf die Anzahl der Mobilfunkverflechtungen der potenziellen Berufspendler und keine Zusammenhänge mit der amtlichen Pendlerrechnung betrachtet werden.

### Diskussion möglicher Modifizierungsansätze der Mobilfunkdatenaufbereitung

Die hier umgesetzten Datenaufbereitungsschritte und Definitionen sowie die daraus resultierenden Variablen in den Mobilfunkdaten haben maßgebliche Einflüsse auf die Anzahl der mobilen Aktivitäten sowie auf die Zusammenhänge mit der amtlichen Pendlerrechnung. Die hohen Korrelationen der übereinstimmenden Pendlerströme zwischen der Pendlerrechnung und den Mobilfunkdaten unterstützen grundsätzlich die Annahme, dass Mobilfunkbewegungen die amtlich ermittelten Pendlerverflechtungen teilweise gut abbilden und theoretisch auch unterstützen könnten. Dennoch wird in allen Analysen und resultierenden Ergebnissen in Abschn. 3 erkenntlich, dass die absoluten Pendlerverflechtungen auf Basis der Mobilfunkdaten systematisch fehlgeschätzt werden. Letzteres wird vor allem durch den Zielkonflikt zwischen ausschließlicher Extrahierung der Berufspendler und dem Informationsgehalt aller verfügbaren mobilen Bewegungsverflechtungen bedingt. Nachfolgend sollen daher Modifizierungsansätze diskutiert werden, wie sowohl eine bessere Darstellung der Berufspendler als auch ein höherer Informationsgehalt in den Mobilfunkdaten aufgrund einer alternativen Datenaufbereitung erreicht werden könnten.

Aufgrund der Abhängigkeit vom Datenanbieter im Rahmen der individuellen Mobilfunkdatenaufbereitung bleiben nur wenige Spielräume, um die Datenaufbereitung optimal an die Zielsetzung anzupassen. Dennoch können anhand der ermittelten Einflüsse auf die Mobilfunkdaten einige Treiber der Falscheinschätzung der potenziellen Pendlerverflechtungen in den Mobilfunkdaten benannt werden.

Ein erstes zu modifizierendes Filterkriterium kann vor allem in der Erhöhung der Verweildauer mobiler Aktivitäten am Zielort gesehen werden. Die hier eingeführte zweistündige Verweildauer führt zu nicht gewünschten Bewegungsverflechtungen, die nicht denen der Berufspendler entsprechen (siehe hierzu Abschn. 3). Die Wahl der Verweildauer ist jedoch abhängig von der Fragestellung. Das bedeutet, dass Teilzeitbeschäftigte eine kürzere Zeitdauer an ihrem potenziellen Arbeitsort verweilen als Vollzeitbeschäftige (siehe hierzu Abb. 5).^34^ Bei der Zielsetzung dieser Arbeit wäre eine Verweildauer von sechs Stunden aufwärts wahrscheinlich zielführender. Bei der Wahl einer höheren Verweildauer muss allerdings darauf geachtet werden, dass die Anzahl mobiler Aktivitäten tendenziell abnehmen und damit die Anzahl an potenziellen Berufspendlern geringer ausfallen wird als diejenigen der Pendlerrechnung und folglich zum oben beschriebenen Zielkonflikt zwischen Genauigkeit und Informationsverlust führt (vgl. Tab. 4; Abb. 6). Im vorliegenden Datensatz würde eine Erhöhung der Verweildauer zu einem extremen Informationsverlust führen, welcher jedoch teilweise abgefangen werden könnte, wenn der Eintritt der gruppierten Mobilfunkaktivitäten in die Zielgemeinde vor 9 Uhr aufgehoben wird.

Für die Unterscheidung zwischen Bewegungsverflechtungen durch Freizeitaktivitäten oder durch Berufstätigkeit ist vor allem der Eintrittszeitpunkt in einen Zielort in den Daten zuständig. Problematisch ist der daraus resultierende Ausschluss von Beschäftigten im Schichtdienst o. ä. durch die bedingte Eintrittszeit. Wird die Verweildauer entsprechend erhöht und gleichzeitig der Eintrittszeitpunkt in eine Zielgemeinde entfernt, wird potenziell der Informationsgehalt in den Mobilfunkdaten gesteigert und vorwiegend mehr Berufspendler in den Daten wiedergegeben. Hierbei sollte beachtet werden, dass das erste und letzte Signal des Tages eines mobilen Endgerätes wieder zwingend in derselben Gemeinde erfasst werden muss. Dies ist bei allen Personenkategorien in den vorliegenden Mobilfunkdaten, mit Ausnahme der Tagespendler, nicht zwangsläufig der Fall.

Dennoch bleibt es aufgrund des 24-Stunden Auswertungszeitraums weiterhin schwierig bspw. Beschäftigte im Nachtdienst zu filtern, solange ihr letztes Signal des Tages nicht am Wohnort registriert wird. Letzten Endes ist eine Abwägung zwischen der detailgetreuen Abbildung der Berufspendler oder anderer Zielgruppen und der Anzahl mobiler Aktivitäten nicht zu vermeiden.

Weiterhin resultieren die Über- und Unterschätzungen der Pendlerströme in den Mobilfunkdaten aus der angewandten Extrapolationsmethodik des Datenanbieters. Eine Extrapolation ist grundsätzlich erforderlich, da der Mobilfunkanbieter Deutsche Telekom ca. ein Drittel des deutschen Mobilfunkmarktes umfasst und damit auch nur ca. 30 % aller deutschen Mobilfunkkundinnen und -kunden abbilden kann.^35^ Für repräsentative Aussagen basierend auf allen Mobilfunkkundinnen und -kunden müssen die resultierenden Mobilfunkdaten entsprechend korrigiert werden. Hierbei erfolgt die Extrapolation auf die Gesamtzahl aller aktiven Mobilfunkgeräte sowie einer anschließenden Hochrechnung auf die Gesamtbevölkerung und ignoriert dabei eine darauffolgende Einteilung der Mobilfunkdaten in verschiedene Sub- bzw. Zielgruppen, wie bspw. die Berufspendler. Folglich kommt es zur Unter- oder auch Überschätzung dieser Zielgruppen, da die Extrapolation die Mobilfunkdaten auf die Grundgesamtheit zwar angleicht bzw. korrigiert, dabei aber eine Korrektur der Daten nach der jeweiligen Zielgruppe außer Acht lässt, wie die Anzahl der Pendlerverflechtungen der Berufspendler in den vorliegenden Mobilfunkdaten demonstrieren. Demzufolge würden nach Zielgruppen extrapolierte Werte zu weniger starken Verzerrungen führen, da diese durch die Kalibrierung auf Ebene der Subgruppen zielorientierter korrigiert werden würden, weshalb eine Extrapolation nach den gewünschten Subgruppen vorzuziehen ist.

Für die effektivere Filterung der Zielgruppen ist weiterhin eine geografische Selektion der Mobilfunkbewegungen anhand von bspw. Gewerbeparks, Bildungseinrichtungen etc. zu prüfen. Dadurch bestünde die Möglichkeit, Bewegungsverflechtungen konkret den Zielorten zuzuordnen und wirksamer von anderen Bewegungsverflechtungen abzugrenzen. Mit dem verfügbaren Mischraster ist dies nicht zu bewerkstelligen, da die Gitterzellen aufgrund ihrer unterschiedlichen Größe diverse Gebäude und damit Personengruppen in den Mobilfunkdaten einfangen. Hierfür bedarf es zudem geeigneter georeferenzierter Daten zu allen Arbeitgebern, Bildungseinrichtungen etc.

Ebenfalls wurden Überlegungen angestellt, um anhand soziodemografischer Merkmale der Mobilfunkkundinnen und -kunden bestimmte Bevölkerungsgruppen zu extrahieren. Beispielsweise könnten anhand der Altersgruppe Bildungspendler von Berufspendlern an Schulen, Universitäten oder anderen Bildungseinrichtungen separiert werden. Da die soziodemografischen Merkmale der Mobilfunkanbieter jedoch nur von Vertragskundinnen und -kunden stammen und diese – je nach Anbieter – stark unterschiedliche Kundenstrukturen aufweisen, ist von einer Nutzung dieser zur Filterung bestimmter Bevölkerungsgruppen abzusehen. Resultierende Ergebnisse würden aufgrund der nicht zu korrigierenden Verzerrungen in den Merkmalsausprägungen zu verfälschten Aussagen führen (siehe hierzu Statistisches Bundesamt 2021b).

Grundsätzlich ist es zudem ratsam einen längeren Auswertungszeitraum zu betrachten als – wie im vorliegenden Fall – drei Monate im Spätsommer/Herbst 2019. Der Wochentag und die Ferienzeiten sind weitere Kriterien, die das Bewegungsverhalten und damit die Anzahl der Bewegungsverflechtungen in den Mobilfunkdaten und die Übereinstimmung mit der Pendlerrechnung beeinflussen. Durch die Exklusion der Schulferien und Feiertage blieben von über 90 Auswertungstagen nur 35 Werktage erhalten. Dies könnte zur Identifizierung von regelmäßigen Pendlerverflechtungen zu wenig sein.

Generell ist eine Nachverfolgung von Bewegungsverflechtungen über 24 h hinaus zu bevorzugen, die jedoch aufgrund von Datenschutzbestimmungen bezüglich des Anonymisierungsverfahrens des Mobilfunkanbieters aktuell nicht verfügbar ist (siehe hierzu Bundesbeauftragte für den Datenschutz und die Informationsfreiheit 2017). Die meisten Modifizierungsansätze zur Korrektur von Unschärfen^36^ bleiben jedoch methodischer Art, auf die nur der Datenanbieter Einfluss hat.

## Fazit und Schlussfolgerung

Im Rahmen des Projekts *Pendler Mobil* wurden in Kooperation mit IT.NRW Analysen zur Bevölkerungsmobilität in NRW auf Basis von Mobilfunkdaten aus dem Netz der Deutschen Telekom durchgeführt. Das Ziel des Projekts war es, Bereiche zu identifizieren, in denen Mobilfunkdaten zu einer Ergänzung der bisherigen Pendlerrechnung beitragen können. Die starken Zusammenhänge zwischen den Mobilfunkdaten und der amtlichen Pendlerrechnung am Beispiel von NRW machen deutlich, dass Mobilfunkdaten im Allgemeinen grundsätzlich das Potenzial haben die amtliche Pendlerrechnung zu unterstützen bzw. zu ergänzen. Besonders eine mögliche Erweiterung durch die kleinräumige Zielorts-Bestimmung, d. h. eine Lokalisierung der am stärksten frequentierten Arbeitsgebiete unter Verwendung der Mobilfunkdaten, bringt einen praktischen Mehrwert für die Optimierung der Infrastruktur oder die Bestimmung von funktionalen Räumen und daraus resultierende Standortentscheidungen von Arbeitgebern. Insgesamt offenbaren sich jedoch noch deutliche Unterschiede zwischen der Anzahl der Pendlerströme aus den vorliegenden Mobilfunkdaten sowie einer flächendeckenden Wiedergabe dieser und der Pendlerrechnung von IT.NRW, die sich besonders auf regionaler Ebene stark auswirken.

Mögliche Gründe für die Über- oder Unterschätzungen der Pendlerverflechtungen liegen unter anderem in den Filterkriterien und Aufbereitungsprozessen der Mobilfunkdaten. Besonders die zweistündige Verweildauer erscheint zu kurz, um nur Berufspendler aus den Mobilfunkdaten zu extrahieren. Hierbei wird ein Zielkonflikt zwischen der Genauigkeit der Abgrenzung von Bewegungsverflechtungen bestimmter Zielgruppen und dem Informationsgehalt in den Mobilfunkdaten provoziert. Die Anzahl mobiler Aktivitäten nach Zielgruppen ist hierbei ein entscheidender Faktor für die Bestimmung der Pendlerverflechtungen. Durch die umgesetzte Extrapolation der Mobilfunkdatenströme auf die Gesamtbevölkerung durch den Datenanbieter entsteht eine Unter- oder auch Überschätzung der Berufspendlerströme im Vergleich zur Pendlerrechnung, da eine Kalibrierung der Verzerrungen nach Subgruppen nicht stattfindet. Dies schlägt sich in der Summe aller Mobilfunkbewegungen im Vergleich zur Pendlerrechnung nieder und folglich in einer nicht plausiblen Abbildung der Pendlerverflechtungen. Von einer zeitlich schnelleren Abbildung von Pendlerströmen oder einer zeitnahen fachlichen Unterstützung der Pendlerrechnung mittels Mobilfunkdaten in Form einer experimentellen Pendlerrechnung muss aufgrund der noch deutlichen Änderungs- bzw. Verbesserungsbedarfe der vorliegenden Mobilfunkdaten aktuell abgesehen werden.

Mögliche Lösungsansätze für eine Verbesserung der Ergebnisse oder auch zielführendere Resultate finden sich einerseits in einer Neuformulierung der Filterkriterien oder auch in der Einbindung einer geografischen Selektion zur Extraktion bestimmter Zielgruppen. Weiterhin bedarf es einer methodischen Änderung bzw. Weiterentwicklung der Extrapolation nach Subgruppen, welche entsprechend eine Kalibrierung der Werte in den Mobilfunkdaten nach ausgewählten Zielgruppen durchführt. Hierdurch würden die Fehlschätzungen der Pendlerströme deutlich minimiert werden. Jedoch sind diese Änderungsbedarfe weiterhin als schwierig umsetzbar anzusehen, solange die Datenaufbereitungsprozesse allein vom Datenanbieter durchgeführt werden und wichtige Methoden und Prozessierungsschritte aufgrund von Geschäftsgeheimnissen bzw. Datenschutzgründen nicht offengelegt werden können. Durch die externe Datenaufbereitung bleiben außerdem die Transparenz sowie die Qualitätseinschätzung der Mobilfunkdaten wie auch der Ergebnisse nicht unberührt.

Dennoch ergeben sich aus den kleinräumigen Zielorts-Bestimmungen vielversprechende Anknüpfungsmöglichkeiten für eine experimentelle kleinräumige Pendlerrechnung bspw. in Form eines Indikators, welcher auf kleinräumige Arbeitsmarktregionen hinweist. Ebenso lassen sich aus den obigen Analysen interessante Anknüpfungspunkte zu einer weiterführenden Untersuchung einer möglichen Darstellung der Bildungspendler und der innergemeindlichen Pendler auf Ebene der Gitterzellen finden, welche in weiteren Machbarkeitsstudien näher betrachtet werden könnten.

Letztlich bezieht sich dieser Artikel nur auf die klassische Form des Pendlerverhaltens, das tägliche Pendeln vom Arbeits- zum Wohnort. Weitere Formen des Pendlerverhaltens sollten in weiteren Forschungsarbeiten fokussierter betrachtet und miteinbezogen werden, wie bspw. die zunehmende Bedeutung der Fern- oder Wochenendpendler (Pütz 2015), die bislang in der amtlichen Pendlerrechnung weniger berücksichtigt werden. Diese können anhand geeigneter Mobilfunkdaten mit modifizierten Definitionen und Datenaufbereitungsmechanismen ggf. herausgearbeitet und für zusätzliche Erweiterungsmöglichkeiten in der amtlichen Pendlerrechnung genutzt werden.

Weitere fachübergreifende Forschungsarbeiten könnten, neben den zuvor beschriebenen methodischen Erweiterungen, eine Verknüpfung von mobilen Bewegungsverflechtungen der potenziellen Berufspendler mit weiteren sozioökonomischen Aspekten zum Gegenstad haben. Bspw. könnten durch eine Betrachtung des Einkommens oder der beruflichen Qualifikation weitere Fragestellungen zum Pendlerverhalten und ihren Ursachen untersucht werden. Zusätzlich ist eine Erweiterung der kleinräumigen Zielorts-Bestimmung zu einem Indikator für die wirtschaftliche Aktivität der identifizierten kleinräumigen Arbeitsregionen, wie in Arhipova et al. (2020), mitzudenken. Für die Erstellung eines solchen Indikators bedarf es weiterer Informationen zum touristischen Verhalten oder der Wohnbevölkerung, die mitunter auch aus geeigneten Mobilfunkdatensätzen gewonnen werden können.

Zuletzt muss betont werden, dass diese Auswertungen nur anhand eines Bundeslandes getätigt wurden. Bundesweite Aussagen zu Pendlerverflechtungen eröffnen möglicherweise weitere bis dahin nicht bekannte Beurteilungen der Mobilfunkdaten. Als langfristiges Ziel wird daher eine Prüfung bundesweiter Erweiterungsmöglichkeiten der amtlichen Pendlerrechnung mit neu aufbereiteten Mobilfunkdaten auf Basis der hier angeführten Modifizierungsansätze angeregt. Für eine bundesweite Repräsentativität und demnach Vervollständigung der Daten werden möglichst Daten aller drei Mobilfunkanbieter in Deutschland benötigt, um so auch Fehleinschätzungen durch die Extrapolationsverfahren zu vermeiden, die durch die Nutzung von Mobilfunkdaten nur eines Netzanbieters entstehen. Die hier vorgestellten Analysen sind demnach ein weiterer Schritt zur Integrierung alternativer Datenquellen in die amtliche Statistik, jedoch bedarf es weiterführender Arbeiten, um letztendlich den Übergang von einer experimentellen hin zu einer amtlichen Statistik zu erreichen. Hierfür ist ein nachhaltiger Zugang zu Mobilfunkdaten unerlässlich. Aufgrund einer fehlenden gesetzlichen Grundlage betreffend den Zugang zu Mobilfunkdaten für Eignungsprüfungen der amtlichen Statistik ist dies jedoch aktuell nicht umsetzbar.

## Supplementary Information





## Footnotes

1 Siehe hierzu die niederländische Mobilitätserhebung* Onderzoek Verplaatsingen in Nederland (OViN)* unter https://www.cbs.nl/nl-nl/onze-diensten/methoden/onderzoeksomschrijvingen/korte-onderzoeksbeschrijvingen/onderzoek-verplaatsingen-in-nederland--ovin--.

2 Unter dem Fachbegriff „Pendler“ werden sowohl Pendlerinnen als auch Pendler verstanden. Aus Gründen der besseren Lesbarkeit wird im Text verallgemeinernd das generische Maskulinum verwendet.

3 Neben der ILO-Definition finden sich weitere Definitionen für den Begriff der Erwerbstätigkeit, auf die hier jedoch nicht weiter eingegangen werden. Interessierte Leserinnen und Leser sind beispielsweise auf das U.S. Bureau of Labor Statistics (https://www.bls.gov/cps/definitions.htm#employed) verwiesen.

4 Insgesamt fließen rund 33 Mio. sozialversicherungspflichtig Beschäftigte in die Beschäftigungsstatistik der BA ein, deren Pendlerverflechtungen anhand der Betriebsnummer des Arbeitgebers und der Anschrift des Versicherten einem Arbeitsort und einem Wohnort bis auf Ebene der Gemeinden zugeordnet werden können (Pütz 2015; Bundesagentur für Arbeit 2020).

5 Siehe hierzu den interaktiven Pendleratlas der BA: https://statistik.arbeitsagentur.de/DE/Navigation/Statistiken/Interaktive-Angebote/Pendleratlas/Pendleratlas-Nav.html. Die Anzahl der sozialversicherungspflichtig Beschäftigten am Arbeits- und Wohnort sowie nach Ein- und Auspendler über Gemeindegrenzen oder auf Kreisebene kann im Statistikportal der Statistischen Ämter des Bundes und der Länder heruntergeladen werden (siehe hierzu auch https://www.destatis.de/DE/Themen/Laender-Regionen/Regionales/Publikationen/Downloads/regiostatkatalog-2019.pdf?__blob=publicationFile).

6 Siehe hierzu den interaktiven Pendleratlas von IT.NRW: https://www.pendleratlas.nrw.de/.

7 Zudem gibt es weitere alternative Pendleratlanten kommerzieller bzw. privater Anbieter wie den Pendleratlas der Mitfahrzentrale für Pendler, die Daten zu Pendlerbewegungen in Deutschland kombiniert (siehe https://www.pendleratlas.de/).

8 Grund hierfür ist die Vollerhebung in der Beschäftigungs- und Personalstandstatistik.

9 Ausgenommen sind Auszubildende mit Ausbildungsvergütung.

10 Angaben zu Bildungspendlern werden vom Statistischen Bundesamt nur als bundesweiter Durchschnitt aus dem Mikrozensus 2016 (Statistisches Bundesamt 2017c) oder wie in Dettmer und Wolf (2018) als Durchschnittswert der Ausbildungspendler für das Bundesland Hessen sowie nach Gemeindegrößenklassen der Wohnsitzgemeinde als mögliche Ergänzung zur Pendlerrechnung veröffentlicht.

11 Die in Abschn. 2 beschriebenen Mobilfunkdaten haben diverse Limitation, wie die repräsentative Angabe der Altersgruppen (siehe bspw. Statistisches Bundesamt 2021b), die es nicht ermöglichen plausible Pendlerverflechtungen von jungen Personengruppen aufzuzeigen (siehe hierzu auch Abschn. 4.2).

12 Städte sind inkludiert und fallen hier vereinfachend unter die Gebietsstruktur der Gemeinden.

13 Unter dem Begriff „Einwohner“ werden im Folgenden sowohl Einwohnerinnen als auch Einwohner verstanden. Aus Gründen der besseren Lesbarkeit wird im Text verallgemeinernd das generische Maskulinum verwendet.

14 Unter dem Begriff „(Tages‑)Tourist“ werden im Folgenden sowohl (Tages‑)Touristinnen als auch (Tages‑)Touristen verstanden. Aus Gründen der besseren Lesbarkeit wird im Text verallgemeinernd das generische Maskulinum verwendet.

15 Eine nachträgliche Aufsummierung der Mobilfunkaktivitäten kann zu Mehrfachzählungen führen, da eine Deduplizierung der Mobilfunkaktivitäten nur auf Basis der ursprünglichen geografischen Ebene durchgeführt wurde. Für einen Abgleich mit der Pendlerrechnung ist es jedoch zwingend erforderlich, die Aktivitäten von der Ebene der Gitterzellen auf die Gemeindeebene zu aggregieren. Verzerrungen oder Unschärfen werden in den Bewertungen der Ergebnisse berücksichtigt.

16 Die Distanz ergibt sich dabei aus: $\text{Distanz}_{i}=\sqrt{\left(dx\right)^{2}+(dy)^{2}}$ wobei $dx=111,3\cdot \cos \left(lat\right)\cdot \left(lon1-lon2\right)$, $lat=\frac{lat1+lat2}{2\cdot 0,01745}$ und $dy=111,3+(lat1-lat2)$. Lat1, lat2, lon1, lon2 entsprechen Breiten- und Längengrade jeder Start- und Zielgemeinde. Die Konstante 0,01745 ergibt sich aus der Umrechnung von Grad in Radiant (Bogenmaß) durch: $1^{\circ}=\frac{\pi }{180rad}$. Der Abstand zwischen zwei Breitenkreisen in km wird durch die Konstante 111,3 und der variable Abstand zwischen zwei Längenkreisen durch $111,3\cdot \cos (lat)$ angegeben (Kompf 2020).

17 Natürlich beinhalten die Quelle-Ziel-Matrizen der Mobilfunkdaten deutlich mehr Wege und damit Verflechtungen, die durch den Nicht-Einbezug weiterer Personenkategorien in diesem Artikel jedoch nicht weiter berücksichtigt werden können.

18 Hierbei handelt es sich vereinfacht um insgesamt 44.559 Auspendlerströme sowie 44.659 Einpendlerströme aus der Pendlerrechnung von IT.NRW.

19 Würde die Korrelation aller Pendlerwege aus der amtlichen Pendlerrechnung mit den Mobilfunkdaten berechnet werden, so würde die Korrelation von 0,85 aller übereinstimmenden Pendlerverflechtungen auf nur noch 0,76 bei den Auspendlerströmen und 0,648 bei den Einpendlerströmen sinken. Grund dafür ist, dass knapp 50 % der amtlich ermittelten Pendlerverflechtungen nicht in den Mobilfunkdaten enthalten sind.

20 Von diesen zusätzlichen Pendelbewegungen gelten 2241 jedoch – gemäß der Pendlerrechnung – als nicht plausibel, da Entfernungen von über 80 km zurückgelegt wurden. Damit verbleiben 4568 potenziell neue Pendlerwege aus den Mobilfunkdaten. Diese beinhalten vorwiegend zusätzliche und von der Anzahl geringere Pendlerverflechtungen (im Durchschnitt 22 Pendler pro Weg) von Städten und Gemeinden mit einer Einwohnerzahl unter 100.000 Einwohnern.

21 Die x‑Achsenlänge beträgt maximal 20.000 gezählte Pendlerbewegungen und die y‑Achsenlänge maximal 15.000 Bewegungen. Beide Achsenlängen resultieren aus den vorliegenden Pendlerströmen in den Mobilfunkdaten und der Pendlerrechnung.

22 Hierbei wird die Anzahl der Mobilfunkdatenströme, die kleiner als die der Pendlerrechnung sind, um ca. −56 % unterschätzt. Dagegen wird die Anzahl der Mobilfunkdatenströme, die größer als die der Pendlerrechnung sind, um ca. 42 % überschätzt.

23 Interessierte Leserinnen und Leser sind für eine vollumfassende visuelle sowie individuelle Betrachtung der Zielorte auf die zum Download verfügbare interaktive Karte „Onlinematerial1.html“ verwiesen. Bei der Karte ist zu beachten, dass hier ausschließlich die Einzugsgebiete auf Ebene der Gitterzellen visualisiert werden.

24 In der Farbabbildung weist die rote Schattierung auf eine höhere Anzahl an Einpendlern hin.

25 © OpenStreetMap-Mitwirkende: Basiskarte und Daten von OpenStreetMap und OpenStreetMap Foundation in Abb. 3 und 4 und Onlinematerial 1.

26 Auch als Chempark, früher als Bayerwerk, bekannt.

27 Siehe hierzu auch die Beispiele in Abb. 3 oder die zum Download verfügbare interaktive Karte „Onlinematerial1.html“.

28 Es wird ein Durchschnittswert aller Aktivitäten über die verbliebenen Auswertungstage aus drei Monaten gebildet, wobei Wochenenden und Feiertage nicht enthalten sind.

29 Weitere Ausführungen zu den methodischen Aspekten bzgl. der Mobilfunkdaten sind in Hadam et al. (2020a) und Statistisches Bundesamt (2019, 2021b) zu finden.

30 Eine Überdispersion tritt unter anderem auf, wenn die Verteilungsannahme der Daten durch eine unbeobachtete Heterogenität in den Daten und dadurch die stochastische Unabhängigkeit der Ereignisse verletzt wird. Dies tritt bspw. bei geclusterten Daten auf (Hilbe 2011, Kap. 7).

31 Der Dispersionsparameter beträgt im vorliegenden Fall 9,563.

32 Vgl. für die praktische Umsetzung in R Venables und Ripley (2002, Kap. 7.4) und Zeileis et al. (2008).

33 Zudem konnte die Überdispersion von 9,563 auf 4,953 reduziert werden.

34 Je nach Fragestellung kann es daher notwendig sein, mehrere Mobilfunkdatensätze zu verwenden.

35 Siehe hierzu Bundesnetzagentur https://www.bundesnetzagentur.de/DE/Sachgebiete/Telekommunikation/Unternehmen_Institutionen/Marktbeobachtung/Deutschland/Mobilfunkteilnehmer/Mobilfunkteilnehmer_node.html.

36 Als Unschärfen werden hier Umgangsformen des Datenanbieters mit ungeeigneten Gegebenheiten in den Mobilfunkdaten verstanden. Darunter fallen Situationen wie das Vorhandensein gemeindeübergreifender Mobilfunkzellen und die Zuordnung mobiler Aktivitäten in eine Gemeinde oder der Umgang mit fehlenden Signalen, wenn bspw. das Mobiltelefon bereits im Untersuchungsgebiet ausgeschaltet wurde und kein letztes Signal in der Endgemeinde mehr erzeugt bzw. registriert wird.
